# Adaptable Fast Relaxing Boronate‐Based Hydrogels for Probing Cell–Matrix Interactions

**DOI:** 10.1002/advs.201800638

**Published:** 2018-07-26

**Authors:** Shengchang Tang, Hao Ma, Hsiu‐Chung Tu, Huei‐Ren Wang, Po‐Chiao Lin, Kristi S. Anseth

**Affiliations:** ^1^ Department of Chemical and Biological Engineering and the BioFrontiers Institute University of Colorado Boulder Jennie Smoly Caruthers Biotechnology Building 3415 Colorado Ave Boulder CO 80303 USA; ^2^ Department of Chemistry National Sun Yat‐sen University No. 70, Lienhai Rd Kaohsiung 80424 Taiwan

**Keywords:** boronate, covalent adaptable networks, hydrogels, stem cells, viscoelasticity

## Abstract

Hydrogels with tunable viscoelasticity hold promise as materials that can recapitulate many dynamic mechanical properties found in native tissues. Here, covalent adaptable boronate bonds are exploited to prepare hydrogels that exhibit fast relaxation, with relaxation time constants on the order of seconds or less, but are stable for long‐term cell culture and are cytocompatible for 3D cell encapsulation. Using human mesenchymal stem cells (hMSC) as a model, the fast relaxation matrix mechanics are found to promote cell–matrix interactions, leading to spreading and an increase in nuclear volume, and induce yes‐associated protein/PDZ binding domain nuclear localization at longer times. All of these effects are exclusively based on the hMSCs' ability to physically remodel their surrounding microenvironment. Given the increasingly recognized importance of viscoelasticity in controlling cell function and fate, it is expected that the synthetic strategies and material platform presented should provide a useful system to study mechanotransduction on and within viscoelastic environments and explore many questions related to matrix biology.

Hydrogels have been widely used as matrices to culture and grow primary cells in 3D with applications in tissue engineering and regenerative medicine.^1^ By incorporating various biochemical, biophysical, and mechanical cues into a single system, hydrogels provide a powerful in vitro model to probe and understand the fundamentals of cell‐niche interactions^2^ and to provide information for testing hypotheses related to developmental and disease‐related processes.^3^, ^4^

Accumulating evidence in the literature suggests that the modulus of synthetic extracellular matrices (ECMs) can have a significant influence on cell function and fate.^2^, ^5^ However, more recently, it has been realized that other time‐dependent mechanical properties, such as viscoelasticity, can also substantially impact cellular responses.^6^, ^7^, ^8^, ^9^ For example, Cooper‐White and co‐workers varied the crosslinking density in poly(acrylamide) gels to control the gels' creep response and showed that the spreading area and proliferation of human mesenchymal stem cells (hMSCs) increased with viscous modulus (or the energy dissipation capacity).^6^ Later, Anseth and co‐workers reported on covalent adaptable networks with hydrazone bonds to prepare poly(ethylene glycol) (PEG) hydrogels with tunable rates of stress relaxation.^7^ Encapsulated C2C12 cells exhibited robust cytoskeletal growth in fast relaxing gels but remained rounded in the slowly relaxing counterparts. Very recently, Chaudhuri et al. controlled the relaxation rate of alginate hydrogels by changing the polymer molecular weights and binding affinity of the calcium chelating domains. Their results suggested that stress relaxation promotes the formation of stress fibers, focal adhesions, nuclear localization of a mechanosensitive transcription regulator yes‐associated protein (YAP), and even osteogenic differentiation of MSCs.^8^, ^9^ Collectively, these studies demonstrate that in addition to matrix modulus, the time‐varying mechanical properties (viscoelasticity) of synthetic ECMs can alter cellular mechanotransduction processes. Because many native tissues exhibit viscoelasticity, viscoelastic synthetic ECMs more closely mimic the dynamic aspects of the cellular microenvironments. This allows experimenters to probe dynamic cell–matrix interactions and provide information that is otherwise difficult to obtain using traditional elastic material systems.

While previous studies have begun to unravel how cells sense their surrounding environment in viscoelastic materials, the field's knowledge of the viscoelastic effect on cellular response remains limited, especially on the short time scales where many important biological processes take place. For instance, on the subcellular level, mechanical signals can propagate from the cytoplasm to the nucleus on the order of seconds, resulting in a concomitant mechanochemical conversion.^10^ However, it is unclear how, and to what extent, these mechanical signals on the small timescales may be integrated to elicit cellular behavior across a broad time spectrum. At the tissue level, many soft tissues such as adipose,^11^ liver,^12^ and brain^13^ relax more than 50% of the imposed stress within 10 s or less. Therefore, synthetic materials with a broader range of fast matrix relaxation kinetics may allow one to probe more connections between time‐dependent material mechanics and cell behavior. Furthermore, based on findings from previous studies,^6^, ^7^, ^8^, ^9^, ^14^ it is reasoned that increasing the rate of matrix relaxation better promotes cell–matrix interactions and potentially amplifies cellular responses to the viscoelastic properties of synthetic ECMs.

From a polymer physics perspective, the rate of stress relaxation, or the characteristic time scale of the viscoelastic properties, in a hydrogel is inherently dictated by the type of physically associative or reversible chemical bonds that create the network crosslinks.^15^, ^16^, ^17^, ^18^ In search of faster relaxation motifs, our investigation focused on dynamic boronate bonds, which started to gain attention in autonomous self‐healing materials,^19^, ^20^ functional bioconjugates,^21^ and complex chemical systems.^22^, ^23^ Boronic esters are often formed between boronic acids and *cis*‐1,2‐diols (**Figure** **1**a). Compared to other reversible bonds or associating motifs, boronates exhibit much faster association and dissociation dynamics.^22^ Moreover, unlike aldehydes that are potentially cytotoxic at high concentrations,^7^ careful selection of the boronic acids and *cis*‐1,2‐diols can circumvent such toxicity concerns. Finally, the formation of boronates occurs with high reaction specificity, with less interference from many functional groups that are present in complex biochemical environment.^22^, ^23^ These features make boronate an attractive candidate for creating fast relaxing materials for studying cellular mechanotransduction processes.

**Figure 1:**
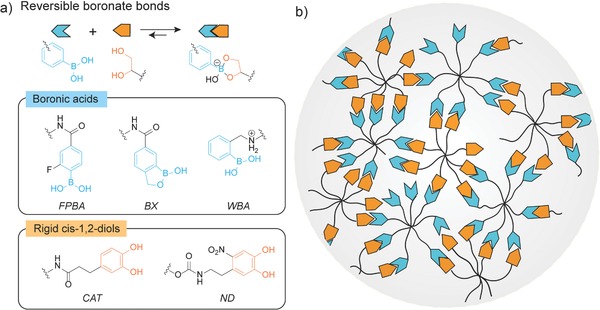
Design of covalent adaptable networks based on dynamic boronate bonds. a) Top: General reaction scheme showing the reversible formation of boronates between boronic acids and *cis*‐1,2‐diols. Bottom: Chemical structures of 2‐fluorophenylboronic acid (FPBA), boroxole (BX), Wulff‐type‐like boronic acid (WBA), catechol (CAT), and nitro‐dopamine (ND) studied in the experiments. b) A schematic of a dynamic network based on boronates from boronic acids and *cis*‐1,2‐diols functionalized octa‐arm PEGs. Unfunctionalized chain ends, dangling chains, and (secondary) loops are depicted as possible defects in the networks.

Although boronate‐based hydrogels have been investigated extensively, few reports demonstrate their use as stable 3D cell culture platforms,^19^, ^24^, ^25^, ^26^, ^27^ likely due to the weak binding strength between boronic acids and diols, particularly under physiological condition. To address this problem, this work rationally selects boronic acid variants and diols that can form dynamic boronate bonds, even in complex biochemical environments (e.g., serum containing media). Specifically, 2‐fluorophenylboronic acid (FPBA), 1‐hydroxy‐1,3‐dihydrobenzo[c][1,2]oxaborole (*m*‐boroxole, BX), and a Wulff‐type *o*‐aminomethylphenylboronic acid (WBA) (Figure 1a) are chosen as the boronic acid derivates. Previous studies have shown that these motifs can form dynamic boronate bonds near physiolocial pH,^27^, ^28^, ^29^ by lowering the p*K*_a_ of boronic acids through an electron withdrawing group, intramolecular B—O coordination, and intramolecular B—N coordination, respectively. In addition, catechol is selected as the diol to attain strong binding between boronic acids and *cis*‐1,2‐diols because the association constants of the catechol‐boronic acid pairs are often two orders of magnitude larger than the complexes formed by conformationally more flexible saccharides.^30^ However, our preliminary experiments using the catechol moieties showed that oxidation of catechol is inevitable over several days, which changes the mechanical properties of the gels with time (Figure S1, Supporting Information). Inspired by a study from Ding et al.,^31^ nitro‐dopamine (ND) with an electron withdrawing nitro group is used to improve the oxidative stability of the catechol. These rational design considerations are expected to tailor the boronate‐based hydrogel properties, rending them particularly suitable for long‐term 3D cell culture.

Polymer networks based on reversible boronate bonds show robust gel formation and a nearly ideal network structure as inferred from rheological characterization. The selected boronic acid and nitrodopamine motifs were covalently conjugated to star‐shaped octa‐arm PEG polymers (*M*_w_ = 20.0 kg mol^−1^, *Đ* = 1.03) via efficient coupling reactions (synthetic details in the Supporting Information). The end group functionalization efficiencies determined by nuclear magnetic resonance (NMR) were about 90% for all the polymers synthesized. When equal parts of the boronic acid and nitro‐dopamine macromolecular gel precursors were mixed in 100 × 10^−3^ m phosphate buffer at pH 7.4, gelation took place within several seconds. Time sweep experiments suggest that gelation was rapid, and the time required to reach the gel point was shorter than the sample preparation time needed to setup the rheological experiment (Figure S3, Supporting Information). Frequency sweep tests show that boronate‐based hydrogels exhibit typical mechanical responses similar to other dynamic covalent adaptable networks^7^ or physically associating networks,^17^, ^18^ including a high‐frequency plateau modulus, a *G*′ − *G*′′ crossover in the intermediate frequency range indicating transition from a solid‐like to a liquid‐like behavior, and a transition to the terminal relaxation behavior where *G*′ and *G*′′ approach to the terminal scaling (*G*′ ∼ ω^2^ and *G*′′ ∼ ω) (**Figure** **2**a and Figure S2, Supporting Information). The mechanical spectra of these dynamic hydrogels were further modeled with stretched exponential functions (fitting details in the Supporting Information). The stretched exponents of the fits are around 0.90, suggesting that the rheological behavior of these materials is nearly Maxwellian and the gels behave nearly as ideal networks, despite the presence of the small defects created by incomplete polymer functionalization and some small length‐scale inhomogeneities that exist the network structure (e.g., loops and clusters). The well‐defined molecular and network structures make these materials attractive and suitable model systems for exploring their structure–property relationships using polymer physics theories.

**Figure 2:**
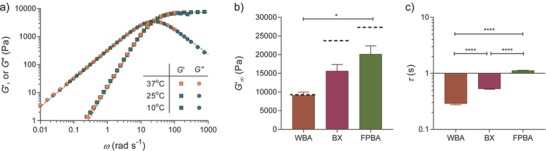
Tunable viscoelastic properties of boronate hydrogels. a) A representative frequency sweep spectrum of boronate hydrogels at 10% (w/v). The rheological master curve with closed symbols is obtained from empirical time‐temperature superposition referenced at 37 °C. The black dashed lines are fits to a stretched exponential function, described in the Supporting Information. b) Comparison of the high‐frequency plateau moduli *G*′_∞_ of gels made from WBA, BX, and FPBA bonding to ND. The black dashed lines show the theoretical moduli estimated from the modified phantom network theory that relates the small molecule equilibrium constant with the network modulus. c) Comparison of the network relaxation time τ = 2π/ω_c_ of different gels plotted on a logarithmic scale. Error bars in (b) and (c) represent standard error of the mean (S.E.M.) from repeated experiments (*n* ≥ 3). Statistical analysis from one‐way analysis of variance (ANOVA) test, * and **** indicating *p* < 0.05 and 0.0001, respectively.

The viscoelastic rheological properties of the boronate‐based hydrogels can be effectively controlled through small molecule design. Two rheological signatures are quantitatively compared to inform the functional group dependence in material properties. The first one is the high‐frequency elastic network modulus *G*′_∞_ (Figure 2b). It is observed that gels synthesized from FPBA motifs give the largest *G*′_∞_, 20.2 ± 3.8 kPa for 10% (w/v) gels, whereas ones made from WBA form the softest materials, with a *G*′_∞_ of 9.1 ± 0.8 kPa. Because these dynamic gels are built on reversible bonds, it is reasoned that the *G*′_∞_ is closely related to the small molecule binding constants. Following the method from Lascano et al.,^22^ UV–vis spectra of ND‐containing solutions were measured in the presence of varying concentrations of different boronic acids (Figure S4, Supporting Information) and the changes in absorption were used to derive the small‐molecule dissociation constants (*K*_D_) of various boronate pairs (Figure S5, method details described in the Supporting Information). Consistent with the hydrogel rheological characterization, the small molecule measurements show that the FPBA‐ND pair is the strongest bond among the three investigated, with a *K*_D_ of 0.23 × 10^−3^ ± 0.02 × 10^−3^ m, and the binding affinity to ND weakens with BX and WBA (Table S1, Supporting Information).

To further elucidate the correlation between the small molecule *K*_D_ and the high‐frequency elastic network modulus *G*′_∞_, phantom network theory was modified to account for the effect of the dissociation/association of the boronates on network mechanics (derivation in the Supporting Information). The theoretical modulus provided by the modified theory matches the experimentally measured results and increases with small molecule *K*_D_ (Figure 2b), further confirming that binding energetics controls the high‐frequency plateau modulus of the dynamic networks. The discrepancy between theory and experiments can be attributed to the presence of molecular defects and network imperfections, such as loops and dangling chains, and non‐negligible network structural inhomogeneity for gels near the overlap concentration.

Variation in the structure of boronic acids not only changes the network modulus but also significantly alters the relaxation behavior of the materials. Here, the relaxation dynamics of boronate gels is characterized by frequency sweeps instead of direct stress relaxation tests to avoid confounding effects from instrument inertia when characterizing fast‐relaxing materials in the short‐time regime. The relaxation time of these dynamic hydrogels is inferred from the *G*′ − *G*′′ crossover frequency, τ = 2π/ω_c_. For all the gels studied here, the relaxation time constants are on the order of 1 s or below (Figure 2c), suggesting fast network relaxation compared to many other dynamic hydrogels reported in literature.^7^, ^9^, ^17^ Interestingly, τ and *G*′_∞_ share the same dependence on the boronic acid structure: the strongest FPBA‐ND bonds show the slowest relaxation and the weakest WBA‐ND bonds show the fastest. For *S*_N_1 type dissociation mechanisms, many studies have concluded that the network relaxation time is governed by the small‐molecule dissociation time.^15^, ^18^ While it is challenging to directly measure the dissociation rate constants of the boronate bonds in aqueous solution, the timescale on which the boronates dissociate is in a similar range as reported by others.^22^, ^32^ Overall, our results demonstrate that the viscoelastic properties of these covalent adaptable networks can be directly related to the properties of the reversible small‐molecule boronate pairs, which provides a foundational basis for the rational design of dynamic gels for cell studies.

A generalizable strategy was further developed to prepare hydrogels that would be stable under cell culture condition, yet still exhibiting significant stress relaxation. While the aforementioned experiments show that the dynamic mechanical properties of gels can be tuned by the chemical structure of the boronate bonds, the fast association–dissociation dynamics renders these materials completely soluble when placed in a sink of cell culture media, often containing excess monosaccharides that facilitate dissociation. Indeed, all the boronate hydrogels completely dissolved in low glucose Dulbecco's modified Eagle's medium (DMEM) within less than 4 h at 37 °C. To overcome the limitation of rapid gel erosion, a secondary, orthogonal crosslinking mechanism was introduced into boronate hydrogels, where the chemistry is based on permanent bonds formed by strain promoted azide‐alkyne cycloaddition (SPAAC) (**Figure** **3**a). When the amount of permanent bonds is above the network percolation threshold, it is expected that the majority of macromolecular gel precursors are covalently bound to a stable, three dimensionally crosslinked network, thus minimizing mass loss over time.

**Figure 3:**
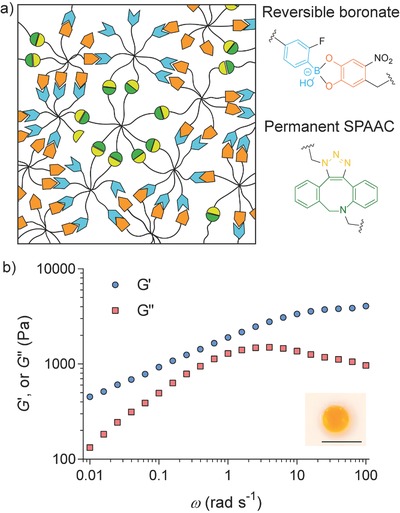
Design of stable hydrogels that exhibit fast relaxation dynamics. a) A schematic showing hybrid networks containing both reversible boronate bonds and permanent SPAAC bonds. b) Representative frequency sweep of hydrogels after being swollen in hMSC growth media (low glucose DMEM, supplemented with 10% fetal bovine serum (FBS), 100 U mL^−1^ penicillin, 100 µg mL^−1^ streptomycin, and 1 µg mL^−1^ fungizone) for 7 d. Measurement was performed at 37 °C. The inset photograph shows gel appearance after being swollen in media for 7 d, demonstrating gel stability. Scale bar 1 cm.

To prove the feasibility of this design concept, a fraction of the end groups of the octa‐arm PEGs were first derivatized into azides and then functionalized with FPBA or ND, respectively (synthetic details in the Supporting Information). The functionality of the azide moieties was confirmed by NMR, showing that each polymer had approximately two azide groups on average. By mixing octa‐arm PEG‐dibenzylcyclooctyne, octa‐arm PEG‐ND/azide, and octa‐arm PEG‐FPBA/azide, gels with hybrid crosslinks formed very quickly, on the order of seconds. Rheological characterization of hybrid gels shows distinctive features compared to the purely adaptable networks (Figure 3b). In the low frequency regime where ω < 0.1 rad s^−1^, *G*′ is always larger than *G*′′ in the hybrid gels and *G*′ approaches a second plateau modulus. On these relatively large timescales, all the reversible boronate bonds are relaxed and therefore do not contribute to the mechanical properties. In contrast, the SPAAC motifs are irreversible, and the hybrid networks maintain its elastic response, even at long times. The network relaxation time, estimated from the frequency at which *G*′′_max_ occurs,^33^, ^34^, ^35^, ^36^ is approximately twice that of the purely adaptable boronate gels. This slight increase in the relaxation time may be due to topological constraints imposed by the permanent network, and similar phenomena have been reported in engineered protein hydrogels with covalent crosslinks^36^ or chain entanglement.^35^ Compared to protein‐polymer hydrogels developed by Dooling and Tirrell,^37^ the boronate‐based hydrogels developed in this work can access similar fast relaxation rates but achieve a larger degree of stress relaxation (over 90% rather than 32–59%, Figure 3b), a characteristic more reminiscent of soft tissues. Furthermore, equilibrium calculations (Supporting Information) suggest that the majority of the boronate bonds retains their adaptability even in the presence of glucose, which only causes a 3% decrease in the total amount of boronate bonds. This is attributed to the dissociation constant of the FPBA‐ND pair, which is approximately two orders of magnitude smaller than that of the FPBA‐glucose pair. This analysis also emphasizes the importance of using ND moieties to attain strong binding to boronic acids. More importantly, these materials are stable under typically used experimental conditions for culturing primary cells and the hMSCs used herein. The initial modulus, relaxation time, dry mass, and volumetric swelling ratio of the gels do not show statistically significant differences over 7 d in media (Figures S6 and S7, Supporting Information). These observations also suggest that the oxidation of ND is negligible in cell culture media within the experimental time window. The absence of degradation and the fast relaxation dynamics make these materials useful for in vitro cell culture in either two or three dimensions (i.e., cells seeded on the surface or encapsulated in the hydrogels).

Hydrogels with the mixed types of crosslinks show exceptional cytocompatibility. Here, hMSCs were encapsulated at a density of 1 × 10^6^ cells mL^−1^ and cultured in growth media for 7 d. For a control, hMSCs were encapsulated at the same density in a purely elastic hydrogel prepared from the SPAAC chemistry alone (formulation in Table S2, Supporting Information), whose elastic modulus (*E* = 14.1 ± 2.7 kPa, assuming a Poisson's ratio of 0.5) closely matched the initial modulus of the hybrid gels (*E* = 16.5 ± 2.4 kPa), despite a small difference in the polymer concentration of gels. Both gels had the fibronectin‐mimicking peptide GRGDS incorporated at a concentration of 3 × 10^−3^ m to promote cell–matrix adhesion. On day 1, cells in both gels showed high viability post encapsulation, 97.0% ± 0.4% and 93.3% ± 2.8% for stress‐relaxing and elastic gels, respectively (**Figure** **4**). On day 7, cells remained over 90% viable in stress‐relaxing gels, and a significant fraction of the cells had spread within these stress‐relaxing materials. In contrast, cell viability decreased to just 81.3% ± 2.0% in the elastic control, and the cells remained rounded. These results show that stress relaxation better maintains cell viability and permits cell–matrix interactions and spreading, even without the presence of any permanent degradation mechanism, such as enzymatically or hydrolytically cleavable linkers. Furthermore, cell proliferation was quantified by directly counting the nuclei density in images of cell‐laden gels. Although the cell density showed a slight increase in the stress‐relaxing gels from day 1 to day 7, the small difference was not statistically significant (Figure S8, Supporting Information). In comparison, the cell density decreased to ≈82% in the elastic gels on day 7 (Figure S8, Supporting Information), which suggests that the aforementioned differences in cell viability are less likely to be related to differences in proliferation.

**Figure 4:**
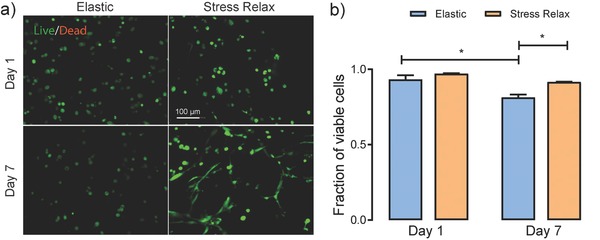
Cytocompatibility of boronate‐based hydrogels. a) Representative maximum intensity projection images of cells in elastic and stress‐relaxing hydrogels on day 1 and day 7. Cells were stained with calcein AM (green, live) and ethidium homodimer (red, dead). Scale bar = 100 µm. b) Quantification of cellular viability. Error bars represent the standard errors of the mean (S.E.M.). Statistical analysis from one‐way ANOVA test, * indicating *p* < 0.05.

Intrigued by the apparent differences observed in the cell morphology between the stress‐relaxing and elastic gels indicated from the calcein staining, the effect of matrix stress relaxation on hMSC morphology and cytoskeletal organization was systematically investigated. Cells were stained for F‐actin with rhodamine phalloidin and for nuclei with 4′,6‐diamidino‐2‐phenylindole (**Figure** **5**a). In the elastic controls, cell volume and sphericity did not change appreciably from day 1 to day 7 (Figure 5b,c). Strikingly, hMSCs were significantly larger in the stress‐relaxing gels and showed even early stages of spreading on day 1, as indicated by the calculated decrease in sphericity (the definition of sphericity is provided in the Supporting Information). During the time course of culture from day 1 to day 7, cell volume continued increasing (Figure 5b and Figure S9, Supporting Information), eventually reaching ≈23 × 10^3^ µm^3^, nearly four times that in the elastic control. The increase in cell volume is reminiscent of previous finding in enzyme‐degradable gels;^38^ however, in the adaptable, stress relaxing gels the changes in cell shape is only permitted through physical remodeling of the surrounding networks. The mechanistic cause for the increase in cell volume might possibly be attributed to an increased density in intracellular content such as proteins and DNAs, and/or mechanical activation of ion channels regulating water influx.^39^ In addition, it is observed that a large fraction of cells extended processes in the stress‐relaxing gels (Figure S10, Supporting Information), reinforcing the fact that the reversible boronate bonds provide a unique means for cell matrix mechanical signaling.

**Figure 5:**
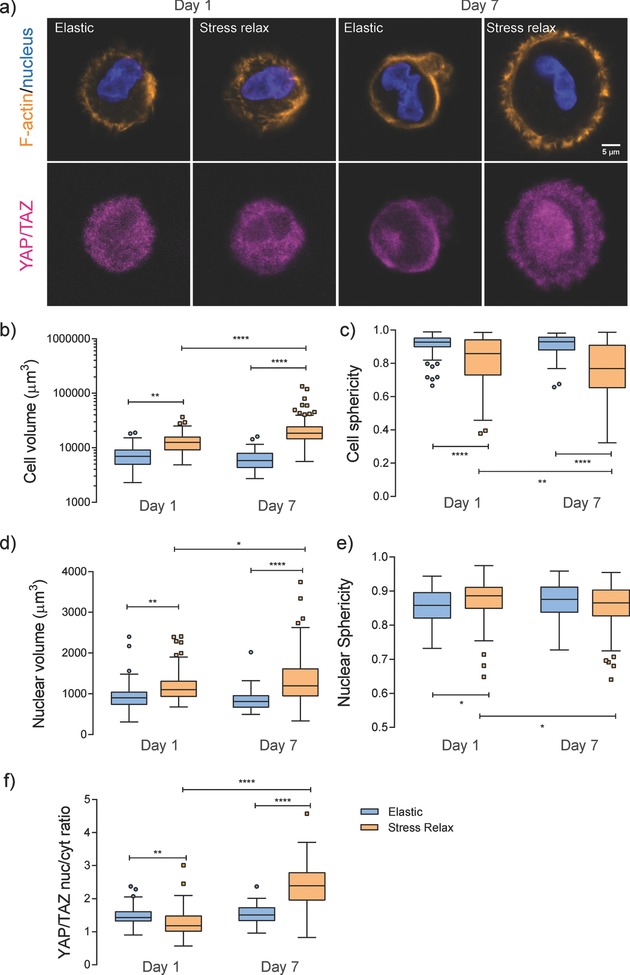
Comparison of cell morphology and YAP/TAZ subcellular localization in elastic and stress‐relaxing gels. a) Representative immunofluorescence staining for F‐actin (orange), nucleus (blue), and YAP/TAZ (magenta) in hMSCs cultured in elastic and stress‐relaxing gels for 1 and 7 d. Images are shown as one cross‐sectional slice of a *z*‐stack with a step size of 1.5 µm. Scale bar = 5 µm. b–f) Quantification of cell volume, cell sphericity, nuclear volume, nuclear sphericity, and YAP/TAZ nuclear to cytosolic intensity ratio at the corresponding condition. Statistical analysis from one‐way ANOVA test, *, **, and **** indicating *p* < 0.05, 0.01, and 0.0001, respectively. Experiments were performed in three replicates per condition, and over 30 cells analyzed per replicate.

To further investigate how cells respond to the fast stress‐relaxing microenvironment, temporal changes in subcellular localization of the transcriptional coactivators YAP/TAZ (yes‐associated proteins and PDZ binding domains) were studied. Recent investigations have shown that YAP/TAZ behaves as cellular rheostats, which largely influence mechanosensing and affect cell function and fate.^2^, ^40^ For hMSCs in elastic hydrogels, YAP/TAZ mainly remained in the cytoplasm throughout the 7 d culture (Figure 5a,f and more representative images in Figure S11, Supporting Information), reminiscent of previous findings in slowly relaxing^9^ or nondegradable gels.^38^ In contrast, significant changes in the YAP/TAZ nuclear to cytoplasmic intensity ratio were observed for cells in the stress relaxing gels. Specifically, YAP/TAZ was primarily in the cytoplasm on day 1, as the case in purely elastic controls; however, quantitative analysis suggested a significant difference between the two gels (Figure 5f). While the mechanistic cause for this finding remains elusive, these results indicate that stress relaxation changes the rate at which cells respond to their surrounding matrices. Even though the relaxation of adaptable gels is fast, on the very short time scales (seconds), significant matrix remodeling is a consequence of numerous cycles of cellular applied stress/strain and matrix relaxation/creep. Therefore, fast viscoelastic mechanics can influence cellular behavior when integrated across a broad time spectrum, even on the long time scales (hours to days) where cell shape and YAP/TAZ subcellular distribution continuously evolve. By day 7, a large population of hMSCs showed nuclear localized YAP/TAZ and the YAP/TAZ nuclear to cytosolic intensity ratio significantly increased (Figure 5a,f). Collectively, these results indicate that YAP/TAZ subcellular localization might be directly related to a cell's ability to remodel its surrounding environment. Interestingly, the aforementioned changes in *cell* volume and sphericity, as well as YAP/TAZ subcellular localization, are associated with changes in *nuclear* volume and shape (Figure 5d,e). This observation implicates a possible role of the nucleus in mechanotransduction in viscoelastic microenvironment, as recently reviewed by Discher and co‐workers^41^ and Szczesny and Mauck.^42^ In addition, correlations were found between cell volume, nuclear volume, and YAP/TAZ nuclear to cytoplasmic intensity ratio (Figure S12, Supporting Information). Given the importance of YAP/TAZ in controlling gene expression, it is expected that these fast‐relaxing gels will likely provide a useful tool to manipulate important mechanical signals for directing MSC fate and current work on this topic is under way.

Last, it should be noted that the experiments above were repeated using hMSCs from a different donor, and all the findings on the effect of viscoelastic properties on stem cell morphology, nuclear morphology, and YAP/TAZ subcellular localization hold (Figure S13, Supporting Information). While admittedly there are some subtle differences in the results between the two biological replicates, these discrepancies are likely due to the differences in donor age and race, but the effect of stress relaxation on cellular responses is general.

In summary, this work has demonstrated that the viscoelastic properties of boronate‐based hydrogels can be readily tuned by changing the small‐molecule structure and properties through rational design. In addition, a robust strategy has been developed to prepare fast stress‐relaxing hydrogels that maintain structural and mechanical properties under typical conditions for culturing primary cells (e.g., hMSCs). While the current study only uses reversible boronate bonds as the stress relaxation motifs, the generality of this approach enables facile incorporation of other dynamic bonds to systematically control the viscoelastic properties of hydrogels over a broad range of timescales. Finally, these fast‐relaxing hydrogels are shown to be cytocompatible, promote cell–matrix interactions, and induce cell spreading and YAP/TAZ nuclear localization. Given the relevance of fast relaxation with many biological processes from subcellular structures to a whole tissue, it is expected that these materials will find use as versatile platforms to further probe the fundamentals of cell–matrix interactions.

## Conflict of Interest

The authors declare no conflict of interest.

## Supporting information

Supplementary
