# Supplementary material for: Adaptable Fast Relaxing Boronate‐Based Hydrogels for Probing Cell–Matrix Interactions
Source: Adv Sci (Weinh). 2018 Jul 26;5(9):1800638. doi: 10.1002/advs.201800638 (PMC6145256; doi:10.1002/advs.201800638)
Supplement: Supplementary file 1 — Supplementary [file ADVS-5-1800638-s001.pdf]

Copyright WILEY-VCH Verlag GmbH & Co. KGaA, 69469 Weinheim, Germany, 2018.

ADVANCED SCIENCE
Open Access

Supporting Information

for *Adv. Sci.,* DOI: 10.1002/advs.201800638

Adaptable Fast Relaxing Boronate-Based Hydrogels for Probing Cell–Matrix Interactions

Shengchang Tang, Hao Ma, Hsiu-Chung Tu, Huei-Ren Wang, Po-Chiao Lin, and Kristi S. Anseth**

Copyright WILEY-VCH Verlag GmbH & Co. KGaA, 69469 Weinheim, Germany, 2016.

Supporting Information

Adaptable Fast Relaxing Boronate-Based Hydrogels for Probing Cell-Matrix Interactions

Shengchang Tang, Hao Ma, Hsiu-Chung Tu, Huei-Ren Wang, Po-Chiao Lin, and Kristi S. Anseth**

Table of Contents:

Supporting Figures Cited in the Main Text Page 3

Supporting Tables Page 16

Materials Page 17

Instrumentation and Characterization Page 17

Synthesis Procedures Page 17

Calculation Details Page 24

Determination of the small-molecule equilibrium constants from titration Page 24

Estimation of the high-frequency plateau modulus of hydrogels from modified phantom network theory Page 25

Boronate equilibrium in PBS and DMEM Page 25

Rheology data processing and analysis Page 26

Volumetric swelling ratio Page 27

Cell studies Page 27

hMSCs Isolation and Cell Culture Page 27

hMSCs encapsulation Page 27

Live/Dead Assay Page 28

Immunostaining Page 28

Image Acquisition and Analysis Page 28

Statistical Analysis Page 29

Additional Supporting Figures Page 30

NMR Spectra Page 32

Supporting Figures Cited in the Main Text

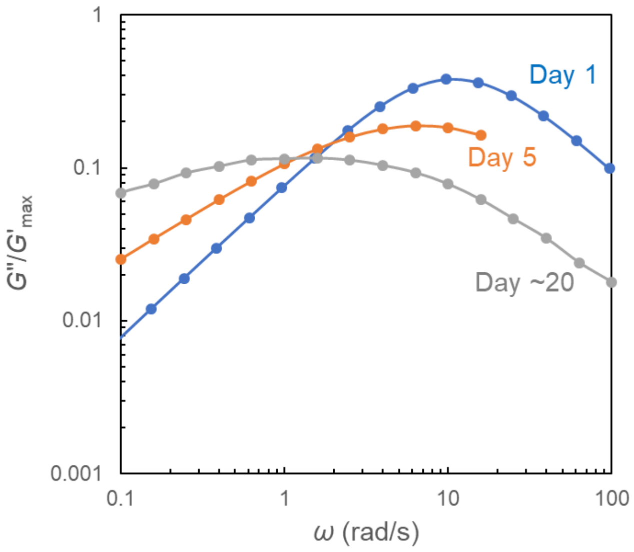

Figure S1. Rheological characterization showing the impact of catechol oxidation on the viscoelastic properties of hydrogels synthesized from octa-arm PEG polymers functionalized with CAT and FPBA. The shift of G''_{max} to the low-frequency end and the decrease in the normalized G'' suggest that gels lose energy dissipation capacity over time and become more elastic, likely due to catechol oxidation. Hydrogel concentration was at 10% (w/v), and the temperature was 37°C.

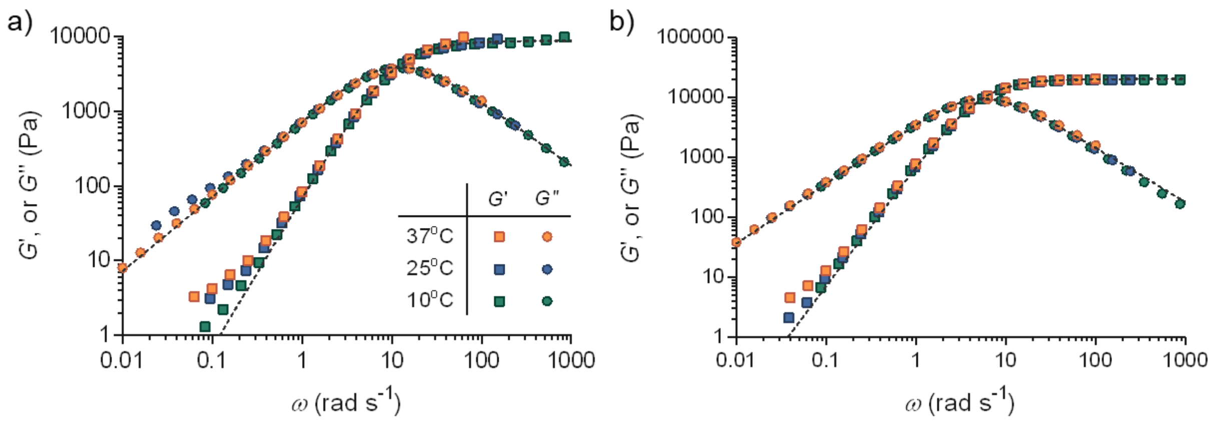

Figure S2. Representative frequency sweep master curves of hydrogels made from (a) BX and (b) FPBA functionalized PEGs with octa-arm PEG-ND. Hydrogel concentrations were at 10% (w/v), and the reference temperature was 37°C.

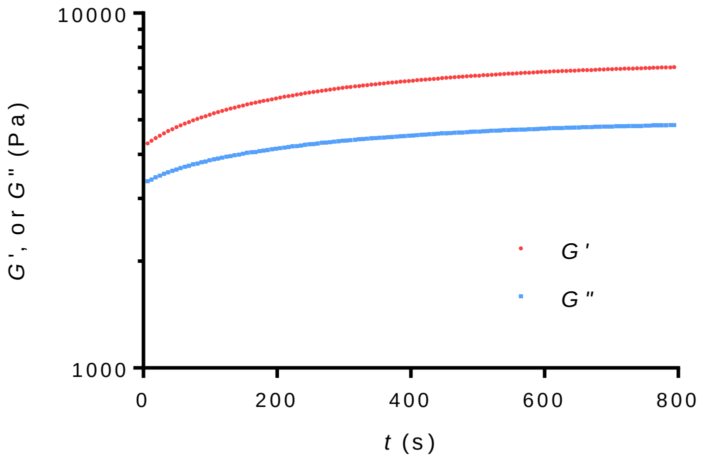

Figure S3. A representative time-sweep spectrum showing that gel evolution as a function of time. After the two macromolecular gel precursors were mixed and time-sweep test was set up, which took approximately 20 s, *G*' had been larger than *G*" at the onset of the experiment. This suggests that gelation of boronate-based hydrogels is very rapid. Gel concentration was at 10% (w/v). Experiment was performed at 37°C, with a fixed angular frequency of 10 rad s^{-1} and 1% strain.

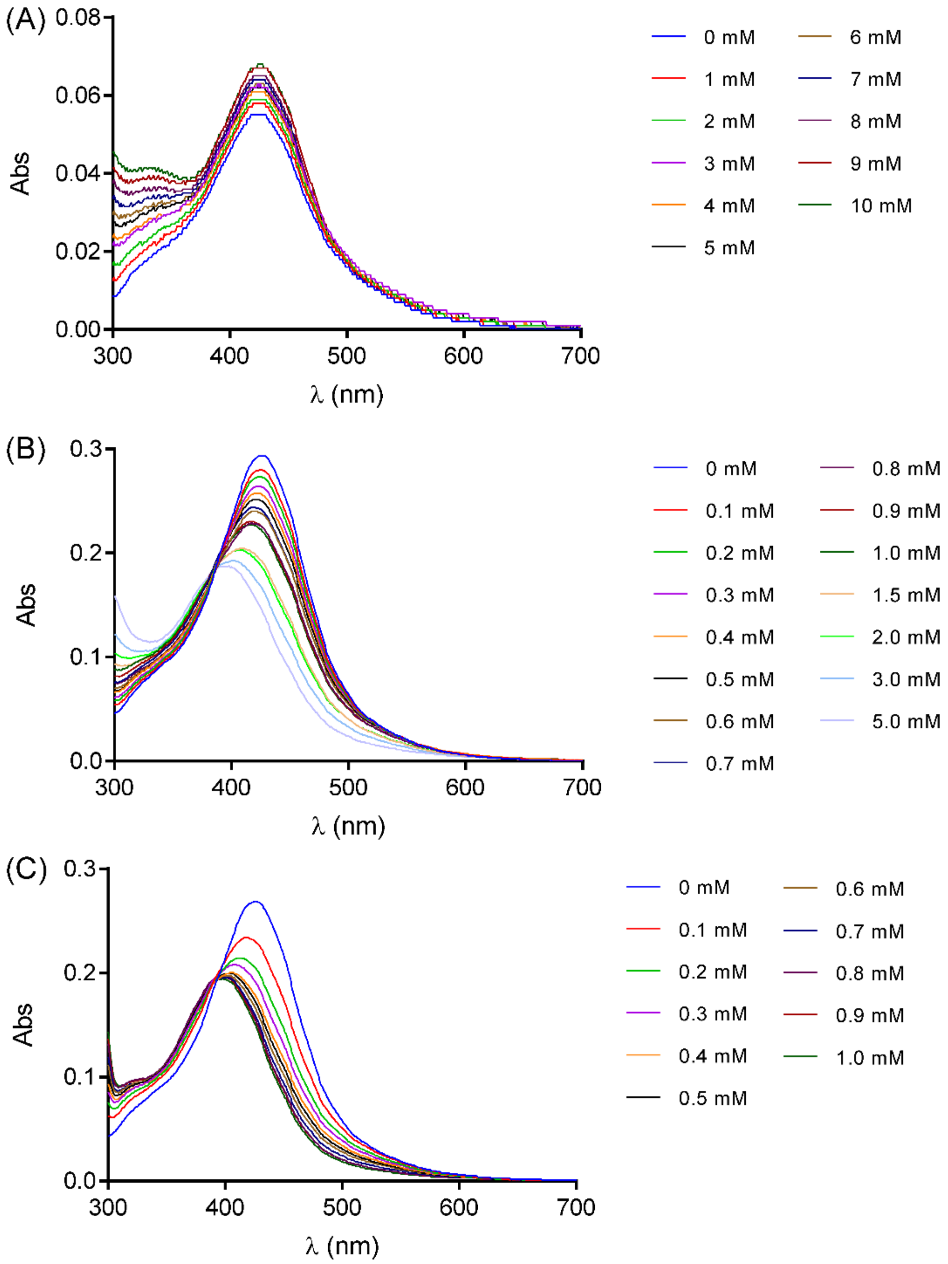

Figure S4. UV-vis spectra of ND in the presence of varying concentration of (A)WBA, (B) BX, and (C) FPBA. The concentrations of ND in (A), (B) and (C) are 8, 40 and 40 μM, respectively. The concentration of WBA, BX and FPBA are shown in the figure legends, in the unit of mM. All the titration experiments were performed at 37°C.

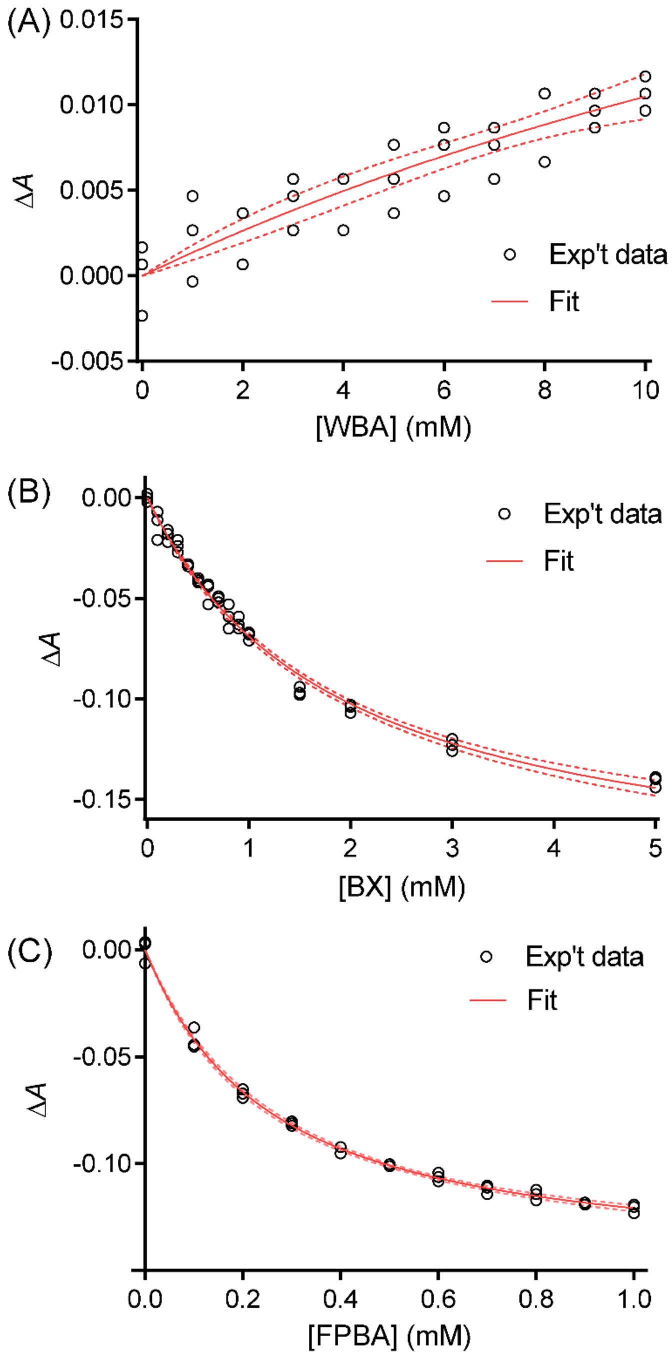

Figure S5. Representative fitting examples showing the relationship of Δ*A* and the concentration of different boronic acids (A) WBA, (B) BX and (C) FPBA. Black open circles are experimental data in triplicate, red solid lines are fits to the 1:1 binding model as described by eq. S1, and the red dash lines are the 95% confidence interval of the fit.

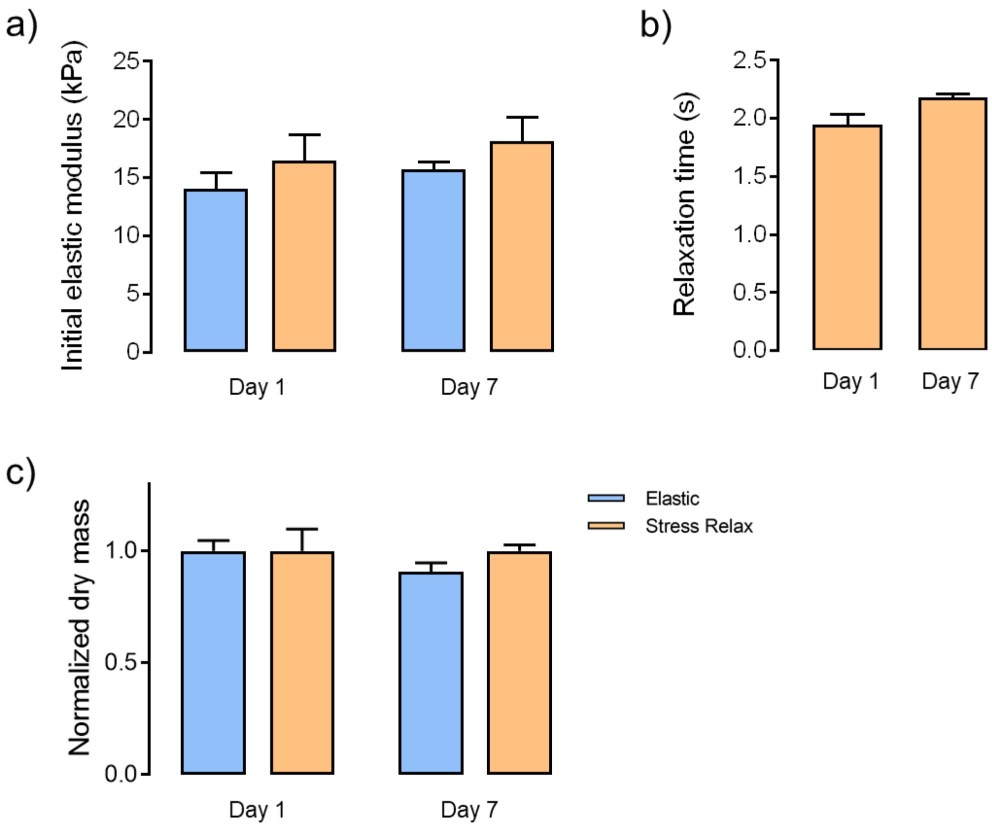

Figure S6. Comparison between the (a) initial elastic modulus, (b) relaxation time, and (c) dry mass for both elastic and stress relaxing gels on days 1 and 7. Error bars represent standard error of the mean (S.E.M., $n = 3$).

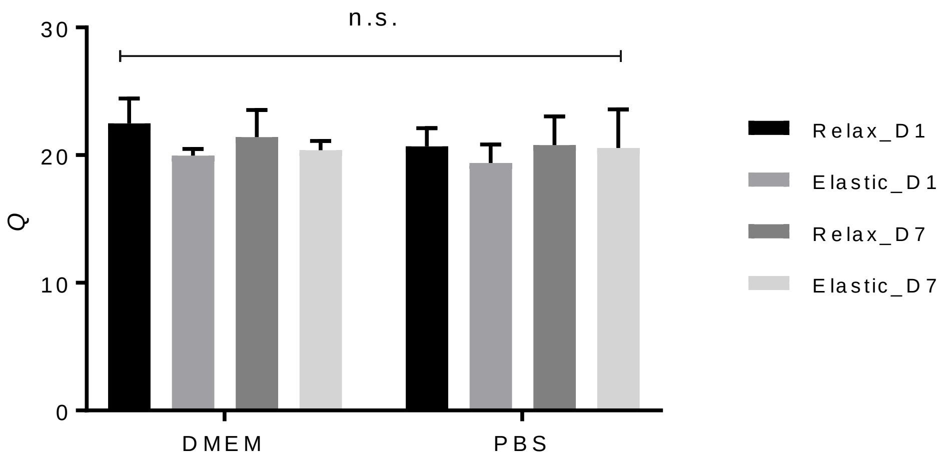

Figure S7. Comparison of the volumetric swelling ratio of elastic and stress-relaxing gels in DMEM and PBS on day 1 and day 7. No statistical differences were observed in all the data sets. Error bars represent standard error of the mean (S.E.M., n = 3).

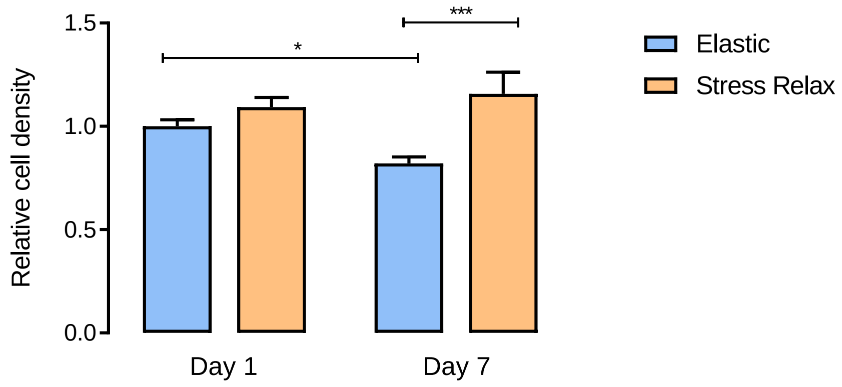

Figure S8. Quantification of hMSC proliferation from direct counting nuclei density in elastic and stress-relaxing gels on day 1 and 7.

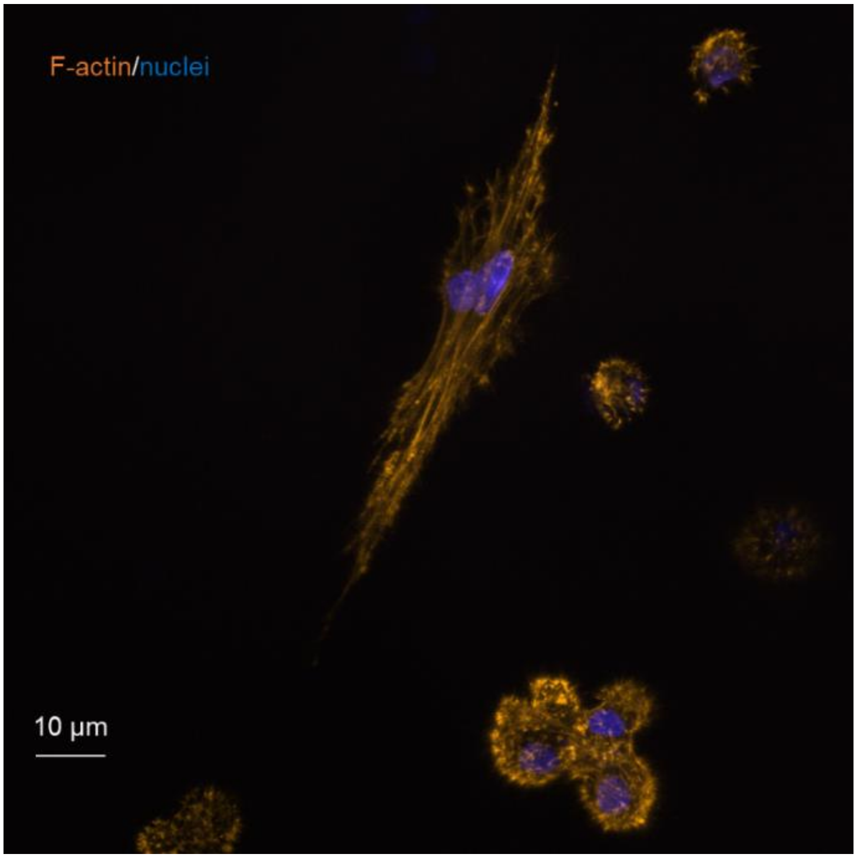

Figure S9. A representative immunofluorescence image (maximum intensity projection) showing spreading of hMSCs encapsulated in stress relaxing gels on day 7.

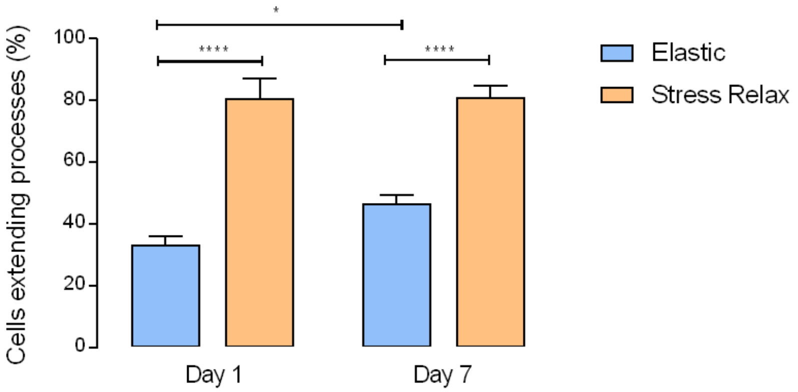

Figure S10. Quantification of the percentage of hMSCs extending processes in elastic and stress-relaxing gels on day 1 and 7.

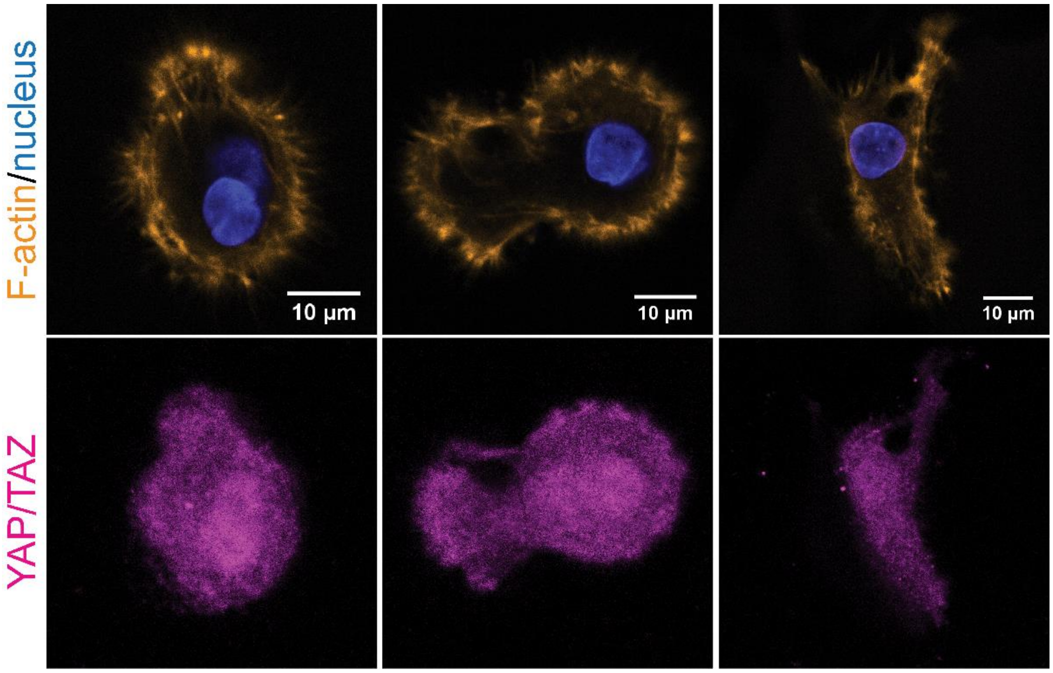

Figure S11. Immunofluorescence images of spread hMSCs cultured in the stress-relaxing hydrogels for 7 days: F-actin (orange), nucleus (blue) and YAP/TAZ (magenta). Note that the scale bars in these images are different.

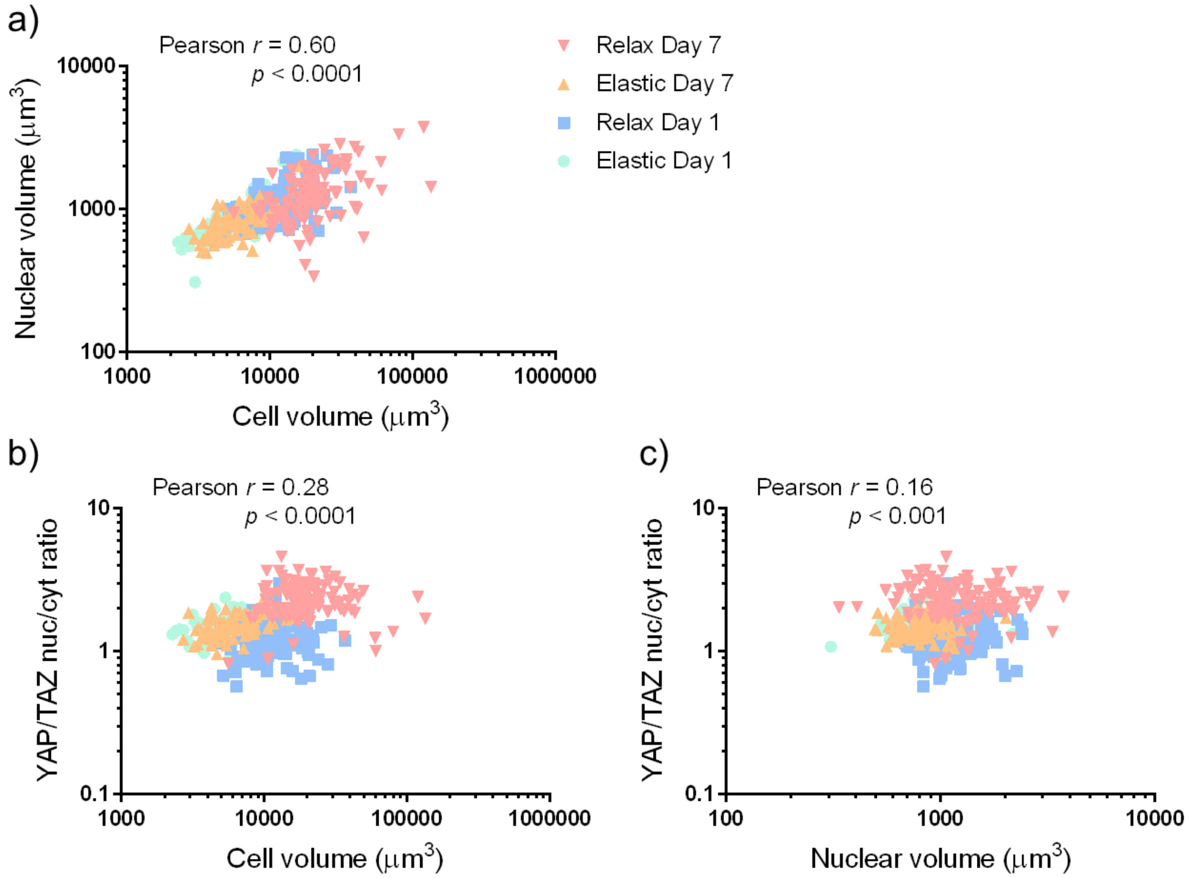

Figure S12. Scatter plots showing correlations between cell volume, nuclear volume, and YAP/TAZ nuclear to cytosolic ratio for hMSCs encapsulated in elastic and stress relaxing gels.

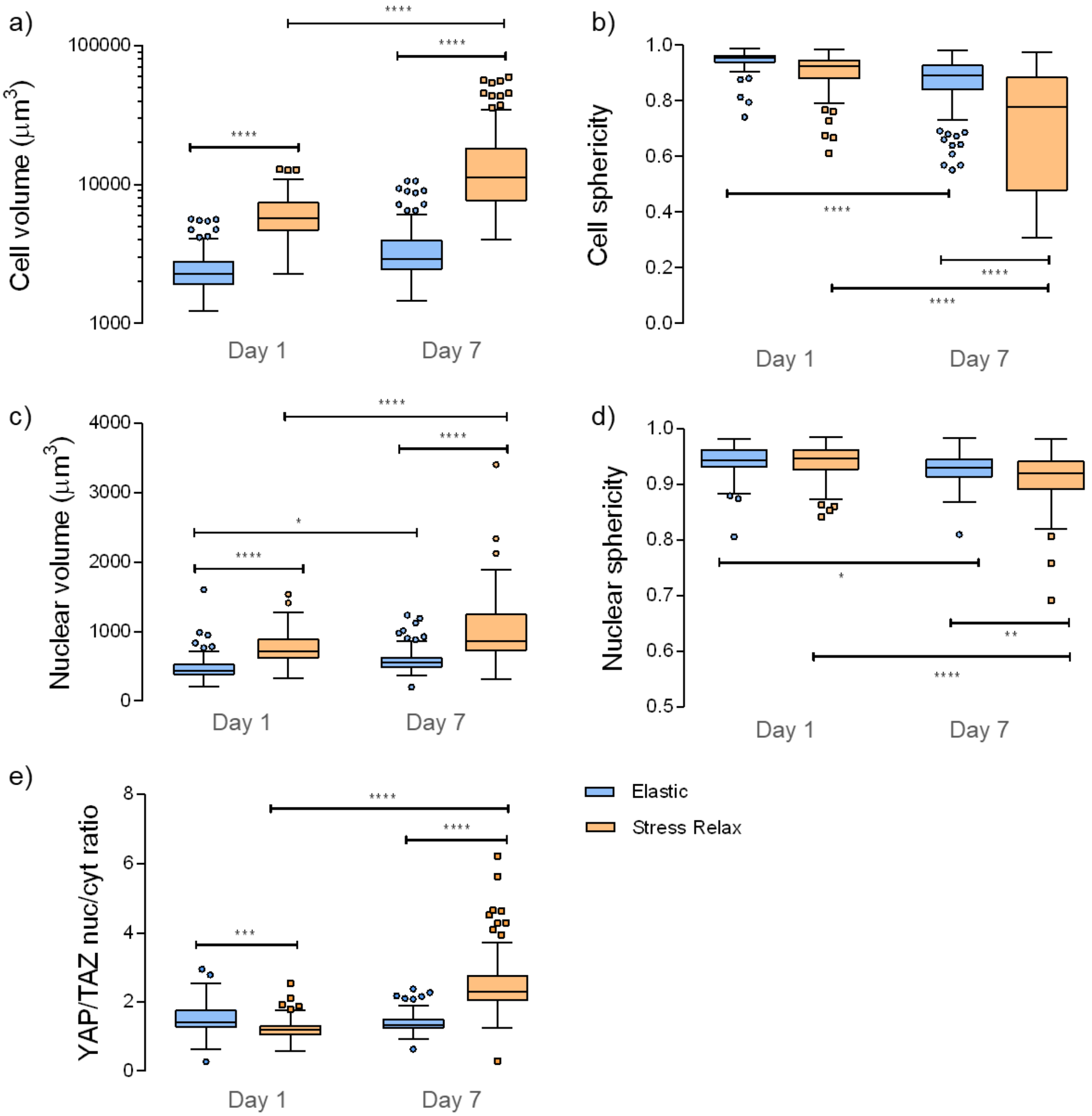

Figure S13. Quantification of (a) cell volume, (b) cell sphericity, (c) nuclear volume, (d) nuclear sphericity, and (e) YAP/TAZ nuclear to cytosolic intensity ratio at the corresponding condition. Statistical analysis from one-way ANOVA test, ** and **** indicating $p < 0.01$ and 0.0001, respectively. Three replicates per condition, and over 30 cells analyzed per replicate. Compared to Figure 5 shown in the main text, the cell and nuclear volumes are smaller in all conditions, perhaps due to the differences in donor race and gender.

Supporting Tables

Table S1. Small molecule dissociation constants (K_D) of ND with different boronic acids in the unit of mM. Uncertainties are 95% confidence intervals of the fits.

Temperature (°C)	WBA*	BX	FPBA
25	14	1.2 ± 0.1	0.12 ± 0.01
37	26	1.8 ± 0.2	0.23 ± 0.02

* The weak binding between WBA and ND yields large uncertainties of the determined K_D, and the value of K_D might be only accurate on the order of magnitude.

Table S2. Final concentration of gel components elastic and stress relaxing gels.

Elastic gel	% (w/v)	Functional group concentration (mM)
Octa-arm PEG-DBCO	3.50	14
Octa-arm PEG-azide	2.75	11
Azido GRGDS		3

Stress relaxing gel	% (w/v)	Functional group concentration (mM)
Octa-arm PEG-DBCO	2.25	9
Octa-arm PEG-azide/FPBA	3.00	3/9
Octa-arm PEG-azide/ND	3.00	3/9
Azido GRGDS		3

Materials

Octa-arm PEG-hydroxyl (M_n = 20 kg mol^{-1}, tripentaerythritol core) and octa-arm PEG-amine (M_n = 20 kg mol^{-1}, hexaglycerol core or tripentaerythritol core) were purchased from JenKem Technology USA. DBCO (dibenzylcyclooctyne) acid, 3-azidopropinoic acid, and azido propylamine were purchased from Click Chemistry Tools. All chemicals for peptide synthesis were purchased from Chem-Impex International Inc. All other chemicals were purchased from Sigma Aldrich or Fisher, and used as received unless otherwise noted.

Instrumentation and Characterization

^{1}H NMR and ^{13}C NMR were recorded on a 400 MHz Bruker AVANCE spectrometer. The residual undeuterated solvent peaks were used for references (7.26 ppm for $CDCl_3$, 4.80 ppm for D_2O, and 5.32 ppm for CD_2Cl_2). The following abbreviations are used to denote multiplicities: s = singlet, d = doublet, t = triplet, q = quartet, m = multiplet, and br = broad. Coupling constants *J* are reported in Hertz (Hz).

UV-vis absorbance spectra were acquired with a Synergy H1 microplate reader (BioTek) at 1 nm resolution. Greiner UV star 96-well plates (flat bottom) were used to ensure accurate readout in the UV range.

Reverse phase high performance liquid chromatography (RP HPLC) purification was performed on a Waters system (2767 sample manager, 2489 UV-vis detector and 2545 binary gradient module) with an XSelect CSH C18 OBD column (5 µm, 30 mm × 250 mm). The mobile phase was a mixture of 0.1% trifluoroacetic acid (TFA) in MilliQ water and HPLC-grade MeCN.

Matrix-assisted laser desorption/ionization time-of-flight (MALDI-TOF) mass spectra were obtained on a Voyager DE mass spectrometer (Applied Biosystems). α-Cyano-4-hydroxycinnamic acid (CHCA) was used matrix. High resolution mass spectra (HRMS) were acquired on a Waters Synapt G2 high definition mass spectrometer.

Rheology experiments were performed on a DHR-3 rheometer (TA instrument) with an 8-mm stainless steel upper plate, and the temperature was controlled by a Peltier bottom plate. Inertial calibration and motor adjustment were performed before each experiment. Frequency sweep experiments were performed at 1% strain (within the linear viscoelastic regime as pre-determined from strain sweep experiments) from 100 to 0.01 rad s^{-1}. For time-temperature superposition, experiments were performed at three different temperatures, namely 10, 25 and 37°C. For measurements of the mechanical properties of swollen hydrogels, adhesive-back sandpaper (600 grit) was applied to both the upper and lower plates. Data with torque values larger than 500 times the minimal torque were reported.

Synthesis Procedures

Synthesis of Azido-Functionalized GRGDS

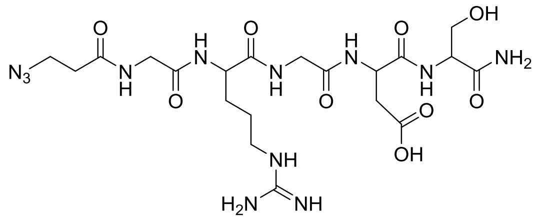

Peptide H-GRGDS-NH_2 was synthesized on rink amide resin via standard Fmoc protection/deprotection chemistry and HATU activation on a Tribute peptide synthesizer (0.5 mmol scale). After deprotection of the N-terminus, the resin was transferred to a peptide synthesis glass vessel and carefully washed with DCM (5 × 10 mL). HATU (760.5 mg, 2.0 mmol), 3-azidopropinoic acid (230.2 mg, 2.0 mmol), DIPEA (700 μL), DMF (7 mL) and DCM (3 mL) were then added to the vessel. After the mixture was stirred at room temperature for 3 h, the resin was washed with DCM (5 × 10 mL). Crude peptides were cleaved with TFA/phenol/triisopropylsilane/water (90:5:2.5:2.5) for 2 h at room temperature and precipitated into cold diethyl ether three times. Crude peptides were further purified by RP-HPLC using a gradient of 0-20% MeCN in water with 0.1% TFA. After lyophilization, pure peptide was obtained as white powder (161 mg, yield: 55%). The purified was confirmed by MALDI-TOF MS (Figure S14), calculated for $C_{20}H_{35}N_{12}O_9$ $[M+H]^+$, 587.3; found 587.4.

Synthesis of Nitrodapamine

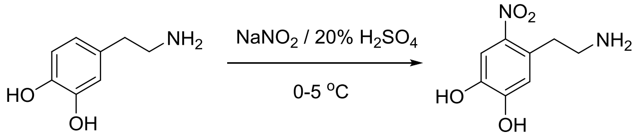

The synthesis of nitrodapamine was performed according to a published procedure[1] with some modifications. Dopamine (500 mg, 2.64 mmol) and $NaNO_2$ (630 mg, 9.13 mmol) were dissolved in MilliQ water (15 mL) and cooled with an ice bath. Then 20% (v/v) sulfuric acid (2.5 mL) was carefully added to the previous solution under vigorous stirring. After ca. 15 min, precipitates were collected by vacuum filtration and washed with cold water three times and then with cold MeOH three times to afford orange solid (400 mg, yield: 51%). ^{1}H NMR (400 MHz, DMSO-d_6): δ 7.44 (s, 1 H), 6.47 (s, 1 H), 3.03 (m, 4 H). HRMS (ESI) m/z calculated for $C_8H_{11}N_2O_4$ $[M+H]^+$, 199.0719; found 199.0734 (Δ = +7.5 ppm).

Synthesis of hydroxyethylaminomethylphenylboronic acid

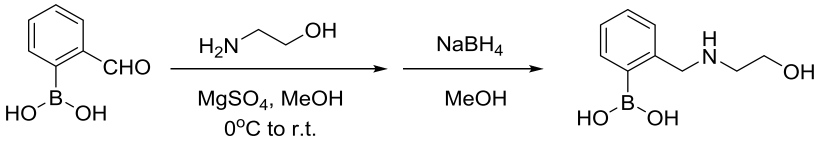

Similar to procedures reported elsewhere[2], to a flame-dried round bottom flask was added 2-formylphenylboronic acid (100 mg, 0.67 mmol), anhydrous $MgSO_4$ (600 mg), and anhydrous MeOH (10 mL). The reaction was cooled to 0°C under an argon atmosphere. Ethanolamine (80.5 μL, 1.33 mmol) was dropwise added to the mixture. The reaction was stirred at 0°C for 30 min and then at room temperature for 3 h. The reaction was again cooled to 0°C, and $NaBH_4$ (100 mg, 2.63 mmol) was added in four portions at 10 min intervals. After being stirred at room temperature for another 2 h, the reaction

was quenched with 1 mL water. The mixture was then filtered through a celite pad and eluted with MeOH. The crude product was further purified by reverse phase HPLC using a Biotage SNAP ultra C18 column with a gradient of 5-100% MeCN in water with 0.1% TFA. Lyophilization of the combined pure fraction afforded a slightly yellow liquid (60 mg, yield: 46%). ^{1}H NMR (400 MHz, MeOD): δ 7.43-7.38 (m, 1 H), 7.26-7.18 (m, 2 H), 7.15-7.10 (m, 1 H), 4.51 (d, *J* = 16.0 Hz, 1 H), 4.01-3.89 (m, 2 H), 3.67-3.58 (m, 1 H), 3.40-3.34 (m, 1 H, overlap with H_2O), 2.94-2.86 (m, 1 H). The structure was also confirmed by gCOSY. ^{13}C NMR (100 MHz, MeOD): δ 142.24, 129.40, 127.42, 126.67, 121.93, 62.13, 54.88, 51.89. Consistent with literature[2], the ipso carbon was not observed. HRMS (ESI) m/z calculated for $C_9H_{15}BNO_3$ $[M+H]^+$, 196.1145; found 196.1163 (Δ = +9.2 ppm).

Synthesis of 1-hydroxy-1,3-dihydrobenzo[c][1,2]oxaborole-6-carboxylic acid

The synthesis of 1-hydroxy-1,3-dihydrobenzo[c][1,2]oxaborole-6-carboxylic acid involved five steps, as detailed below.

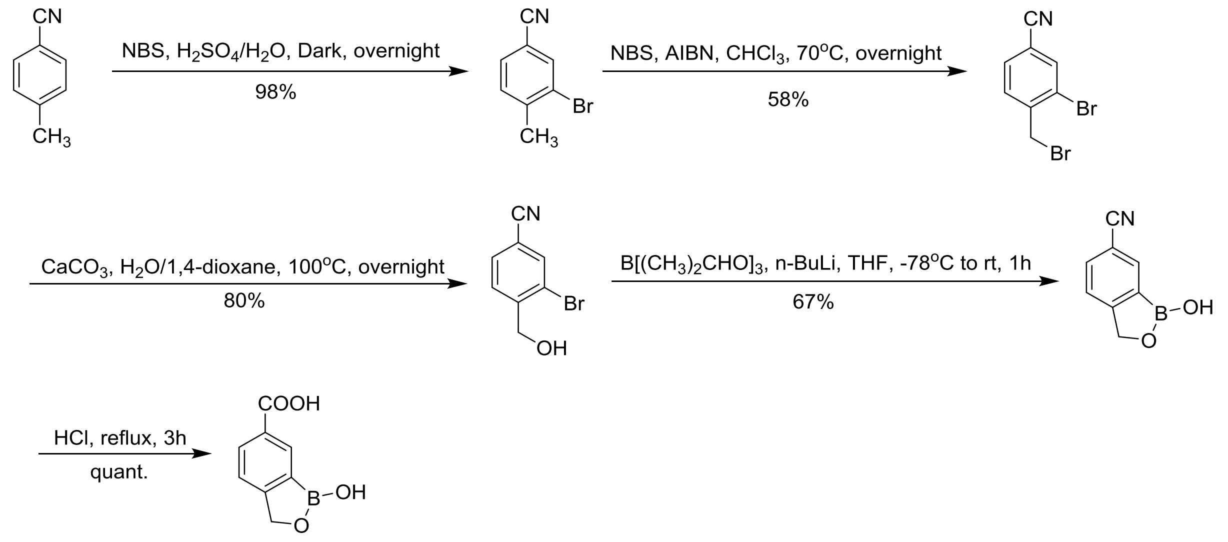

a) Synthesis of 3-bromo-4-methylbenzonitrile[3]

To a stirred solution of 4-methylbenzonitrile (5.0 g, 42.7 mmol) in H_2O (35 mL) and sulfonic acid (35 mL), *N*-bromosuccinimide (7.60 g, 42.7 mmol) was added drop wise in dark and the mixture was allowed to react at 25°C for 12 h. The reaction mixture was quenched by sodium bicarbonate (100 mL) and then washed with ethylether (3 × 50 mL). The collected organic layer was then dried over anhydrous $MgSO_4$, filtered and concentrated to afford the 3-bromo-4-methylbenzonitrile as a white solid (8.17 g, 98%). Mp 84-87°C; ^{1}H NMR (300 MHz, $CDCl_3$) δ 7.81 (s, 1H), 7.49 (d, *J* = 7.9 Hz, 1H), 7.33 (d, *J* = 7.8 Hz, 1H), 2.46 (s, 3H); ^{13}C NMR (75 MHz, CDCl3) δ 144.2, 135.7, 131.5, 131.0, 125.4, 117.7, 111.5, 23.6; HRMS (FAB): calculated for C_8H_6BrN $[M]^+$: 194.9684; found: 194.9681 (Δ = -1.5 ppm).

b) Synthesis of 3-bromo-4-(bromomethyl)benzonitrile[4]

To a stirred solution of 3-bromo-4-methylbenzonitrile (7.13 g, 36.4 mmol) in chloroform (73 mL), *N*-bromosuccinimide (7.38 g, 41.5 mmol) was added, and the mixture purged under nitrogen system for 30 min. Then, azobisisobutyronitrile (418 mg, 2.55 mmol) was added to the reaction mixture and refluxed for 12 h. The reaction mixture was then quenched by brine (100 mL) and extracted with dichloromethane (3 × 50 mL). The collected organic layer was then dried over anhydrous $MgSO_4$, filtered and concentrated

to afford the crude mixture and then purified by flash column chromatography to afford the desired product as a white solid (5.82 g, 58%). Mp 92-95°C; ^{1}H NMR (300 MHz, $CDCl_3$) δ 7.88 (s, 1H), 7.61 (d, *J* = 8.3 Hz, 1H), 7.57 (d, *J* = 7.9 Hz, 1H), 4.58 (s, 2H); ^{13}C NMR (75 MHz, $CDCl_3$) δ 142.6, 136.7, 131.9, 131.6, 125.0, 117.0, 114.1, 31.7; HRMS (EI): calculated for $C_8H_5Br_2N$ $[M]^+$: 272.8789; found: 272.8787 (Δ = -0.7 ppm).

c) Synthesis of 3-bromo-4-(hydroxymethyl)benzonitrile[5]

To a stirred solution of 3-bromo-4-(bromomethyl)benzonitrile (5.82 g, 21.2 mmol) in water (106 mL) and 1,4-dioxane (70 mL) co-solvent system, calcium carbonate (921 mg, 9.20 mmol) was added and reacted at 100°C for 12h. The reaction mixture was diluted with ethyl ether (50 mL) and washed by ddH_2O. The collected organic layer was then dried over anhydrous $MgSO_4$, filtered and concentrated to afford the crude mixture and then purified by flash column chromatography to afford the desired product as a white solid (3.6 g, 80%). mp 148-150 ^{o}C; ^{1}H NMR (300 MHz, $CDCl_3$) δ 7.82 (s, 1H), 7.69 (d, *J* = 7.9 Hz, 1H), 7.64 (d, *J* = 8.3 Hz, 1H), 4.81 (d, *J* = 5.8 Hz, 2H), 2.02 (t, *J* = 5.8 Hz, 1H); ^{13}C NMR (75 MHz, $CDCl_3$) δ 145.7, 135.7, 131.4, 128.6, 122.1, 117.6, 112.7, 64.4; HRMS (EI): calculated for C_8H_6BrNO $[M]^+$: 210.9633; found: 210.9637 (Δ = +1.9 ppm).

d) Synthesis of 1-hydroxy-1,3-dihydrobenzo[c][1,2]oxaborole-6-carbonitrile[6]

To a stirred solution of 3-bromo-4-(hydroxymethyl)benzonitrile (1.0 g, 4.72 mmol) in THF (47 mL), triisopropyl borate (2.18 mL, 9.43 mmol) was added to the mixture at -78°C. Then, n-butyl lithium (6.63 mL, 10.61 mmol) was added drop wise. Then, the reaction mixture was allowed to react at 25°C for 1 h. The reaction mixture was quenched by HCl (50 mL) and then washed with ethyl acetate (3 × 50 mL). The collected organic layer was then dried over anhydrous $MgSO_4$, filtered and concentrated to afford the crude mixture and then purified by flash column chromatography to afford the desired product as a yellow solid (503 mg, 67%). Mp 220-222 ^{o}C; ^{1}H NMR (300 MHz, MeOD) δ 7.97 (s, 1H), 7.78 (d, *J* = 8.1 Hz, 1H), 7.57 (d, *J* = 7.9 Hz, 1H), 5.14 (s, 2H); ^{13}C NMR (75 MHz, MeOD) δ 160.0, 135.5, 135.3, 123.8, 120.1, 112.4, 72.5; HRMS (EI): calculated for $C_8H_6BNO_2$ $[M]^+$: 159.0492; found: 159.0494 (Δ = +1.2 ppm).

e) Synthesis of 1-hydroxy-1,3-dihydrobenzo[c][1,2]oxaborole-6-carboxylic acid[6]

A solution of 1-hydroxy-1,3-dihydrobenzo[c][1,2]oxaborole-6- carboxylic acid (510 mg, 3.21 mmol) in HCl solution (37%, 20 mL) was refluxed at 100°C for 3 h. The precipitate was washed with ice water three times and then dissolved in MeOH. The collected MeOH layer was then dried over anhydrous $MgSO_4$, filtered and concentrated to afford the desired product as a white solid (589 mg, quant.). Mp 227-229°C; ^{1}H NMR (300 MHz, MeOD) δ 8.30 (s, 1H), 8.13 (dd, *J* = 8.0, 0.9 Hz, 1H), 7.48 (d, *J* = 7.9 Hz, 1H), 5.13 (s, 2H); ^{13}C NMR (75 MHz, MeOD) δ 169.9, 160.0, 133.2, 132.9, 131.0, 122.4, 72.2; HRMS (ESI): calculated for $C_8H_6BNO_4$ $[M-H]^-$: 177.0359; found: 177.0355 (Δ = -2.2 ppm).

Synthesis of Octa-Arm PEG-Azide

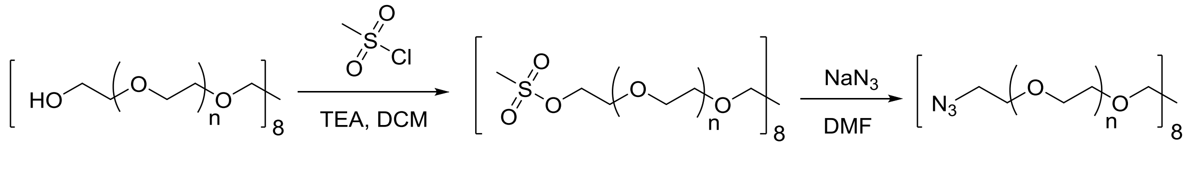

The synthesis of octa-arm PEG-azide was performed according to a published procedure[7] for the synthesis of tetra-arm PEG-azide. In the first step, octa-arm PEG-hydroxyl (5.00 g, 0.25 mmol, M_n = 20 kg mol^{-1}) was dried at 80 °C under vacuum overnight. After the compound was cooled down to room temperature, anhydrous DCM (150 mL) was added to completely dissolve the polymers. Then triethylamine (TEA, 2.50 mL, 18 mmol) and mesyl chloride (1.24 mL, 16 mmol) were added to the solution. The reaction was stirred under argon overnight. The mixture was then diluted with 300 mL DCM and washed with brine (4 × 50 mL). The organic phase was dried over Na_2SO_4, filtered, and concentrated under vacuum. Polymers were recovered by precipitation into diethyl ether twice to give slightly yellow solid (5.01 g, yield: 97%). ^{1}H NMR (400 MHz, $CDCl_3$): 4.38-4.34 (m, 16 H), 3.87-3.21 (m, 1912 H), 3.06 (s, 24 H). The end group conversion determined from ^{1}H NMR was 95%.

In the second step, octa-arm PEG-mesylate (2.50 g, 0.125 mmol), NaN_3 (0.39 g, 6 mmol) and anhydrous DMF (25 mL) were added to a round bottom flask. The reaction was stirred at 60°C for 2 days. The solution was then dialyzed again water extensively (MWCO 8 kDa), and then lyophilized to give white (2.38 g, yield: 95%). The complete disappearance of the mesyl peaks suggests that the end group conversion was quantitative.

Synthesis of Octa-Arm PEG-DBCO

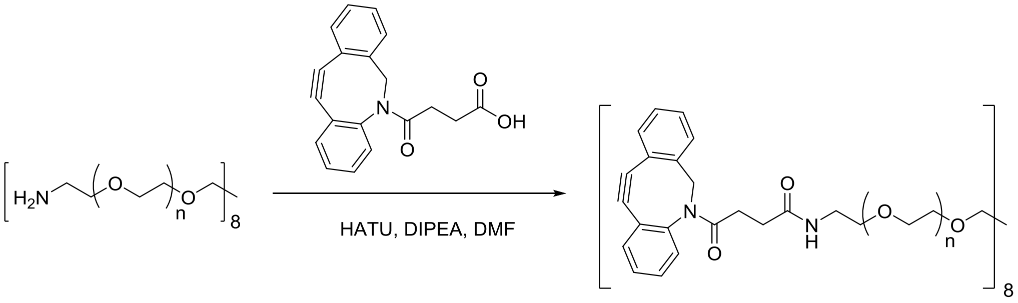

To a reaction vial equipped with a stir bar was added DBCO acid (91.5 mg, 0.3 mmol), HATU (114.1 mg, 0.3 mmol) and anhydrous DMF (1.5 mL). The solution was stirred at room temperature for 10 min and then transferred to a separate flask containing octa-arm PEG-amine HCl salts (500 mg, 25 µmol, M_n = 20 kg mol^{-1}, tripentaerythritol core). The vial was rinsed with DMF (1.5 mL), and the solvent was added to the flask. DIPEA (279 µL, 1.6 mmol) was added to the mixture. The reaction was stirred under argon for 1.5 days at room temperature. Polymers were precipitated into cold diethyl ether, air dried, dialyzed against water extensively (MWCO 8 kDa), and then lyophilized to give yellow power (487 mg, yield: 87%). ^{1}H NMR (400 MHz, CD_2Cl_2): δ 7.68-7.63 (m, 8 H), 7.55-7.50 (m, 8 H), 7.46-7.24 (m, 48 H), 6.22-6.07 (br, 8 H), 5.16-5.08 (d, J = 16.0 Hz, 8 H), 3.89-3.21 (m, 1976 H), 2.80-2.66 (m, 8 H), 2.44-2.29 (m, 8 H), 2.19-2.06 (m, 8 H), 1.97-1.84 (m, 8 H, overlap with H_2O). The end group conversion determined from ^{1}H NMR was 93%.

Synthesis of Octa-Arm PEG-2-Fluorophenylboronic Acid

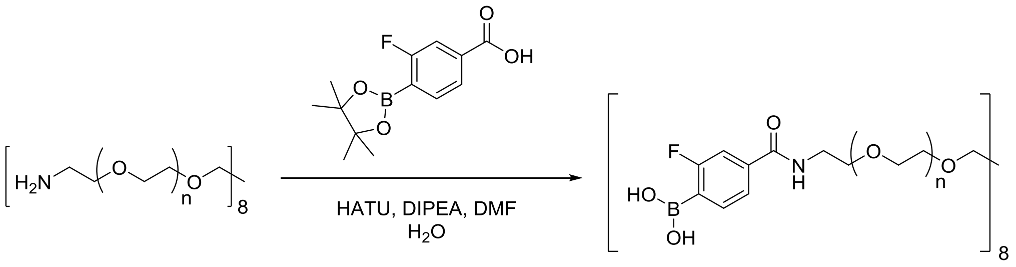

To a reaction vial equipped with a stir bar was added 4-carboxy-2-fluorophenyl boronic acid pinacol ester (133 mg, 0.5 mmol), HATU (190 mg, 0.5 mmol), and anhydrous DMF (1.5 mL). The solution was stirred at room temperature for 10 min and then transferred to a separate flask containing octa-arm PEG-amine HCl salts (500 mg, 25 µmol, M_n = 20 kg mol^{-1}, hexaglycerol core). The vial was rinsed with DMF (1.5 mL), and the solvent was added to the flask. DIPEA (279 µL, 1.6 mmol) was added to the mixture. The reaction was stirred under argon for 1.5 days at room temperature. Polymers were precipitated into cold diethyl ether, air dried, dialyzed against water extensively (MWCO 8 kDa), and then lyophilized to give white power (514 mg, yield: 96%). ^{1}H NMR (400 MHz, $CDCl_3$): δ 7.85 (t, *J* = 7.0 Hz, 8 H), 7.57 (dd, *J* = 1.2 and 8.0 Hz, 8 H), 7.51 (dd, *J* = 1.2 and 8.0 Hz, 8 H), 7.11 (t, *J* = 4.8 Hz, 8 H), 6.09 (s, 16 H), 3.80 (t, *J* = 4.8 Hz, 16 H), 3.79-3.48 (m, 2104 H), 3.45 (t, *J* = 4.8 Hz, 16 H). It is noted that the pinacol ester protecting groups were completely removed during dialysis. The end group conversion determined from ^{1}H NMR was 86%.

Synthesis of Octa-Arm PEG-Boroxole

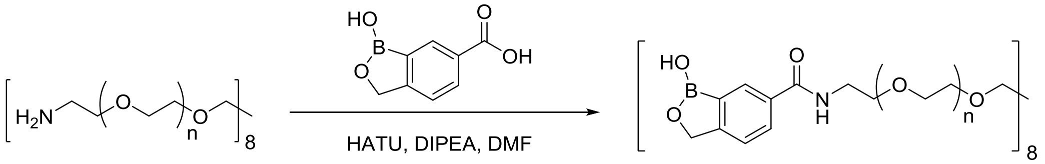

To a reaction vial equipped with a stir bar was added boroxole carboxylic acid (45 mg, 0.25 mmol), HATU (96 mg, 0.25 mmol), and anhydrous DMF (1.5 mL). The solution was stirred at room temperature for 10 min and then transferred to a separate flask containing octa-arm PEG-amine HCl salts (316 mg, 15.8 µmol, M_n = 20 kg mol^{-1}, hexaglycerol core). The vial was rinsed with DMF (0.5 mL), and the solvent was added to the flask. DIPEA (200 µL, 1.15 mmol) was added to the mixture. The reaction was stirred under argon for 2 days at room temperature. Polymers were precipitated into cold diethyl ether twice, air dried, dialyzed against water extensively (MWCO 8 kDa), and then lyophilized to give white power (296 mg, yield: 88%). ^{1}H NMR (400 MHz, D_2O): δ 8.02 (s, 8 H), 7.85 (d, *J* = 8.0 Hz, 8 H), 7.49 (d, *J* = 8.0 Hz, 8 H), 5.06 (s, 16 H), 3.82 (s, 16 H), 3.78-3.50 (m, 2065 H), 3.46 (s, 16 H). The end group conversion determined from ^{1}H NMR was 88%.

Synthesis of *Octa-Arm PEG-o-Aminomethylphenylboronic Acid*

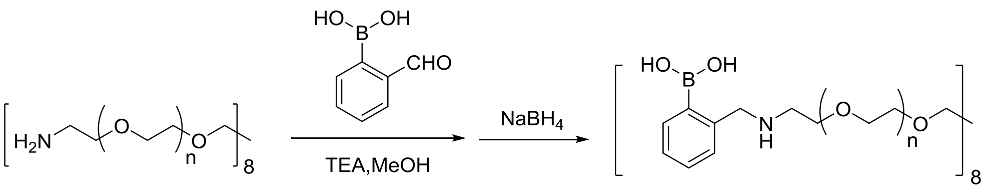

The synthesis of octa-arm PEG-o-aminomethylphenylboronic acid was performed according to a published procedure[8]. To a reaction vial equipped with a stir bar was added octa-arm PEG-amine HCl salts (250 mg, 12.5 μmol, M_n = 20 kg mol^{-1}, hexaglycerol core), 2-formylphenylboronic acid (37.5 mg, 0.25 mmol), triethylamine (TEA, 200 μL, 1.44 mmol), and anhydrous MeOH (2.5 mL). The reaction was stirred under argon at room temperature for 3 days. After this time, the mixture was cooled to 0°C with an ice bath, and $NaBH_4$ (80 mg, 2.13 mmol) was added to the mixture in four portions at 10 min intervals. The reaction was purged with Ar(g) again and left at room temperature for another 20 h. The reaction mixture was then dialyzed against water extensively (MWCO 8 kDa), filtered and lyophilized to give white powder (235 mg, yield: 92%). ^{1}H NMR (400 MHz, D_2O): δ 7.44-7.12 (m, 32 H), 4.10-3.94 (m, 15 H), 3.87-3.39 (m, 1814 H), 3.05 (br, 12 H). The end group conversion was determined to be nearly 100% from ^{1}H NMR analysis.

Synthesis of Octa-Arm PEG-p-Nitrophenylcarbonate

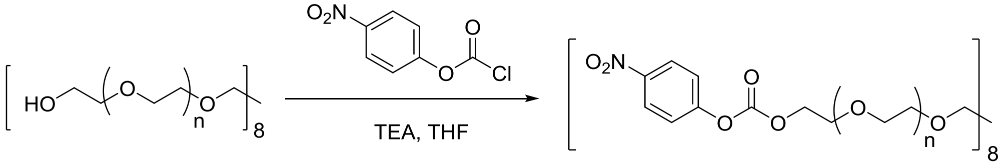

Octa-arm PEG-hydroxyl (2.00 g, 0.1 mmol, M_n = 20 kg mol^{-1}) was dried at 80 °C under vacuum overnight. After the compound was cooled down to room temperature, anhydrous THF (60 mL) was added to completely dissolve the polymers. Then *p*-nitrophenyl chloroformate (1.29 g, 6.4 mmol) and TEA (1.00 mL, 7.2 mmol) were added to the solution. The reaction was stirred under argon at room temperature for 2 days. The purified product was obtained by precipitation into cold diethyl ether three times and drying under vacuum to yield off-white solid (1.95 g, yield: 98%). ^{1}H NMR (400 MHz, $CDCl_3$): δ 8.27 (d, *J* = 9.2 Hz, 16 H), 7.38 (d, *J* = 9.2 Hz, 16 H), 4.42 (m, 16 H), 3.80 (m, 32 H), 3.77-3.21 (m, 2002 H). The end group conversion was determined to be greater than 91% from ^{1}H NMR analysis.

Synthesis of Octa-Arm PEG-nitrodopamine

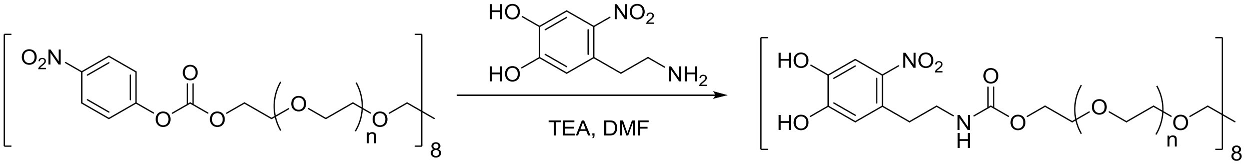

To a round bottom flask was added octa-arm PEG-*p*-nitrophenylcarbonate (250 mg, 12.5 μmol), nitrodopamine (59.5 mg, 0.3 mmol), TEA (86 μL), and DMF (2 mL). The reaction mixture was stirred under argon at room temperature for 2 days, and then dialyzed against water extensively (MWCO 8 kDa) and lyophilized to afford yellow powder (223 mg, yield: 83%). ^{1}H NMR (400 MHz, D_2O): δ 7.58 (s, 8 H),

6.74 (s, 8 H), 4.05 (s, 16 H), 3.80 (s, 16 H), 3.75-3.49 (m, 2046 H), 3.49-3.19 (m, 48 H), 2.97 (m, 16 H). The end group conversion was determined to be 89% from ^{1}H NMR analysis.

Synthesis of Octa-Arm PEG-Azide/Fluorophenylboronic acid

To a reaction vial equipped with a stir bar was added 3-azidopropionic acid (4.3 mg, 37.5 μmol), HATU (14.3 mg, 37.5 μmol), and anhydrous DMF (1.5 mL). The solution was stirred at room temperature for 10 min and then transferred to a separate flask containing octa-arm PEG-amine HCl salts (500 mg, 25 μmol, M_n = 20 kg mol^{-1}, tripentaerythritol core). The vial was rinsed with DMF (0.5 mL), and the solvent was added to the flask. DIPEA (279 μL, 1.60 mmol) was added to the mixture. The reaction was stirred under argon overnight at room temperature. Polymers were recovered by dialysis against water and then lyophilization. By ^{1}H NMR analysis and TNBS (2,4,6-trinitrobenzene sulfonic acid) assay, on average 2 out of the 8 arms were functionalized into azide.

In the second step, 4-carboxy-2-fluorophenyl boronic acid pinacol ester (100 mg, 0.38 mmol), HATU (142 mg, 0.38 mmol) were dissolved in anhydrous DMF (2 mL). The solution was stirred at room temperature for 10 min and then transferred to a separate flask containing the previously obtained polymers and DIPEA (200 μL, 1.15 mmol). The reaction was stirred under argon overnight at room temperature. Polymers were recovered by dialysis against water and then lyophilization. ^{1}H NMR analysis shows that the other 6 arms were functionalized with fluorophenylboronic acid.

Synthesis of Octa-Arm PEG-Azide/Nitrodopamine

To a round bottom flask was added octa-arm PEG-*p*-nitrophenylcarbonate (250 mg, 12.5 μmol), azidopropylamine (1.84 μL, 18.8 μmol), DIPEA (70 μL), and DMF (2 mL). The reaction mixture was stirred under argon at room temperature for 1.5 days. Then nitrodopamine (59.5 mg, 0.30 mmol) was added to the reaction mixture, which was purged with argon and left to stir at room temperature of 1.5 days. The solution was then dialyzed against water extensively (MWCO 8 kDa) and lyophilized to afford yellow powder. ^{1}H NMR analysis suggests that the ratio of azide to nitrodopamine in the final polymers was 1:3.

Calculation Details

Determination of the small-molecule equilibrium constants from titration

The small-molecule boronate ester equilibrium constants were determined by UV-vis titration following published procedures[9]. All measurements were performed in 100 mM phosphate buffer (pH 7.4), identical to the condition used for preparing hydrogels. The dissociation constants K_D were calculated based on the following equation:

$$\Delta A = \frac{\Delta A_{max} K_D}{2[\mathrm{ND}]_0}\left\{\left(1+\frac{[\mathrm{B}]}{K_D}+\frac{[\mathrm{ND}]_0}{K_D}\right)-\sqrt{\left(1+\frac{[\mathrm{B}]}{K_D}+\frac{[\mathrm{ND}]_0}{K_D}\right)^2-4\frac{[\mathrm{B}]}{K_D}\frac{[\mathrm{ND}]_0}{K_D}}\right\} \quad \text{(S1)}$$

where $\Delta A = A - A_0$, the difference in absorbance at 425 nm, $[\mathrm{ND}]_0$ is the initial concentration of nitrodopamine, [B] is the initial concentration of different boronic acids. The range of [B] varies to

maximize the changes in absorbances for different boronic acids. In eq. S1, the two fitting parameters are K_D and ΔA_{max}. All fittings were performed in MATLAB. Representative titration and fitting results are shown in Figure S9 and S10. The derived values of K_D are tabulated in Table S1.

Estimation of the high-frequency plateau modulus of hydrogels from modified phantom network theory

The phantom network theory estimates the network elastic modulus G_{ph} as follows,

$$G_{\mathrm{ph}} = vkT(1-2/f) \tag{S2}$$

where v is the number density of the elastically effective network strands, kT is the thermal energy, f is the junction functionality (f = 8 for octa-arm PEG polymers). In the case of reversible networks with crosslinks based on dynamic association and dissociation of boronate esters, v is affected by the equilibrium constants of the boronates. By assuming that the small-molecule boronates and the polymer-conjugated boronates have the same equilibrium constants K_D, the fraction of associated bonds ζ under each hydrogel condition can be calculated.

$$K_{\mathrm{D}} = \frac{[\mathrm{B}][\mathrm{ND}]}{[\mathrm{B\text{-}ND}]} = \frac{f_{\mathrm{B}}[\mathrm{P}](1-\zeta)\cdot[\mathrm{P}](f_{\mathrm{ND}}-f_{\mathrm{B}}\zeta)}{[\mathrm{P}]f_{\mathrm{B}}\zeta} = \frac{(1-\zeta)[\mathrm{P}](f_{\mathrm{ND}}-f_{\mathrm{B}}\zeta)}{\zeta} \tag{S3}$$

where [P] is the concentration of polymers at equilibrium (note that both boronic acid and nitro-dopamine functionalized polymers are at the same concentration), and f_B and f_{ND} are the number of functional groups on boronic acid and nitro-dopamine functionalized polymers, respectively ($f_B \leq f_{ND} \leq 8$ for the polymers synthesized in this work).

The fraction of associated bonds ζ can be solved from eq S3, which gives,

$$\zeta = \frac{1}{2}\left[\left(1+\frac{1}{r}+\frac{K_{\mathrm{D}}}{[\mathrm{P}]f_{\mathrm{B}}}\right) - \sqrt{\left(1+\frac{1}{r}+\frac{K_{\mathrm{D}}}{[\mathrm{P}]f_{\mathrm{B}}}\right)^2 - \frac{4}{r}}\right] \tag{S4}$$

where r is the stoichiometric imbalance, defined as r = f_B/f_{ND}.

In addition,

$$v = [\mathrm{P}]\zeta N_{\mathrm{A}} \tag{S5}$$

where N_A is the Avogadro constant.

By combining eqs S2, S4 and S5, the high-frequency plateau modulus of the reversible gels is estimated to be

$$G_{\mathrm{ph}} = \frac{3}{8}[\mathrm{P}]RTf_{\mathrm{B}}\left[\left(1+\frac{1}{r}+\frac{K_{\mathrm{D}}}{[\mathrm{P}]f_{\mathrm{B}}}\right) - \sqrt{\left(1+\frac{1}{r}+\frac{K_{\mathrm{D}}}{[\mathrm{P}]f_{\mathrm{B}}}\right)^2 - \frac{4}{r}}\right] \tag{S6}$$

Boronate equilibrium in hybrid gels in PBS and DMEM

Here, the concentration of FPBA-ND boronate pair is calculated in the following two scenarios, one in PBS without the presence of glucose, and the other in DMEM for cell studies, with a glucose concentration of approximately 5 mM.

As shown in Table S2, the concentrations of FPBA and ND were both 9 mM in the hybrid gels containing two types of crosslinks. Therefore, the equilibrium between FPBA and ND in PBS can be described as

$$K_{\mathrm{D}} = \frac{[\mathrm{FPBA}][\mathrm{ND}]}{[\mathrm{FPBA\text{-}ND}]} = \frac{([\mathrm{FPBA}]_0 - [\mathrm{FPBA\text{-}ND}])([\mathrm{ND}]_0 - [\mathrm{FPBA\text{-}ND}])}{[\mathrm{FPBA\text{-}ND}]} \quad \text{(S7)}$$

where the concentration of the FPBA-ND complex is the only unknown in eq S7. Solving eq S7 gives

$$[\mathrm{FPBA\text{-}ND}] = 7.67\ \mathrm{mM}\text{, without glucose} \quad \text{(S8)}$$

For the hybrid gels immersed in DMEM, two equilibrium equations should be solved simultaneously, one for FPBA-ND and the other for FPBA-glucose, which are

$$K_{\mathrm{D,1}} = \frac{[\mathrm{FPBA}][\mathrm{ND}]}{[\mathrm{FPBA\text{-}ND}]} = \frac{([\mathrm{FPBA}]_0 - [\mathrm{FPBA\text{-}ND}] - [\mathrm{FPBA\text{-}Glucose}])([\mathrm{ND}]_0 - [\mathrm{FPBA\text{-}ND}])}{[\mathrm{FPBA\text{-}ND}]} \quad \text{(S9)}$$

$$K_{\mathrm{D,2}} = \frac{[\mathrm{FPBA}][\mathrm{Glucose}]}{[\mathrm{FPBA\text{-}Glucose}]} = \frac{([\mathrm{FPBA}]_0 - [\mathrm{FPBA - ND}] - [\mathrm{FPBA\text{-}Glucose}])([\mathrm{Glucose}]_0 - [\mathrm{FPBA\text{-}Glucose}])}{[\mathrm{FPBA\text{-}Glucose}]} \quad \text{(S10)}$$

where $K_{\mathrm{D,2}}$ is estimated to be 20 mM, according to the work by Yan *et al*.[10]

The two unknowns in eq S9 and S10 are [FPBA-ND] and [FPBA-Glucose]. Solving S9 and S10 gives

$$[\mathrm{FPBA\text{-}ND}] = 7.54\ \mathrm{mM}\text{, with glucose} \quad \text{(S11)}$$

$$[\mathrm{FPBA\text{-}Glucose}] = 0.28\ \mathrm{mM} \quad \text{(S12)}$$

Therefore, the presence of low concentration glucose only introduced a 3% difference in the concentration of adaptive FPBA-ND pairs.

Rheology data processing and analysis

The detailed method of fitting the rheology data to a stretched exponential function is described in detail elsewhere[11]. Briefly, a continuous relaxation spectrum $H(\tau)$ was first constructed based on the dynamic moduli in the master curve obtained from time-temperature superposition. Then, the relaxation modulus $G(t)$ was calculated based on the relaxation spectrum using the following equation:

$$G(t) = \int_{-\infty}^{\infty} H(\tau)\exp(-t/\tau)\,d\ln\tau \quad \text{(S13)}$$

The experimentally derived stress relaxation curve was then fit to a stretched exponential function, or the Kohlrausch-Williams-Watts (KWW) model, which provides an empirical formula to mathematically capture the time dependent stress relaxation modulus of the covalent adaptable networks:

$$G(t) = G_0 \exp\left[-\left(t/\tau_{\mathrm{KWW}}\right)^{\beta}\right] \quad \text{(S14)}$$

where τ_{KWW} is the KWW time constant. β is the stretched exponent ranging from 0 to 1, characterizing the heterogeneity of the network. When $\beta = 1$, the stretched exponential function reduces to the Maxwell model.

Finally, the dynamic moduli were computed using a Fourier Transform[12]:

$$G'(\omega) = \omega\int_0^{\omega} G(t)\sin \omega t dt \quad \text{(S15)}$$

$$G''(\omega) = \omega\int_0^{\omega} G(t)\cos \omega t dt \quad \text{(S16)}$$

Swelling ratio measurements

The volumetric swelling ratios (Q_{V}) of elastic and stress-relaxing gels in both DPBS and DMEM were measured following a previously published procedure. Briefly, gels were prepared in a 0.5 mm thick rubber mold between two glass slides pre-treated with Sigmacote® siliconizing reagents. After gelation, the gels were carefully removed from the molds and transferred into DPBS or DMEM. Buffer was refreshed the first day and then every other day. One day 1 and 7, the wet weights (W_{w}) of hydrogels were measured, and then lyophilized in pre-weighed vials to enable measurements of dry weights (W_{d}) of hydrogels. The volumetric swelling ratio is calculated as

$$Q_V = 1 + \frac{\rho_{\mathrm{p}}}{\rho_s}\left(\frac{W_{\mathrm{w}}}{W_d} - 1\right) \quad \text{(S17)}$$

where ρ_{p} is the density of PEG[13] (taken as 1.23 g cm^{-3}) and ρ_{s} is the solvent density (1.00 g cm^{-3} for DPBS, and 1.01 g cm^{-3} for DMEM used in cell culture).

Cell Studies

hMSC isolation and cell culture

Human mesenchymal stem cells (hMSCs) were isolated from human bone marrow (Lonza) by their adhesion to tissue culture polystyrene (TCPS) plates using established protocols[14]. Isolated hMSCs were frozen down in 80% FBS and 20% DMSO (denoted as P1 hMSCs). P1 hMSCs were used in all experiments and cultured in standard growth media (low-glucose Dulbecco's modified Eagle's medium, supplemented with 10% FBS, 100 U mL^{-1} penicillin, 100 µg mL^{-1} streptomycin, and 1 µg mL^{-1} fungizone) at 37°C and 5% CO_2 unless otherwise noted.

hMSCs from two different donors were separately used in this study, one from an 18-year-old black female, and the other from a 32-year-old Hispanic female.

hMSC encapsulation

P1 hMSCs were plated onto TCPS in growth media with 1 ng mL^{-1} basic fibroblast growth factor (bFGF) for approximately 60 h prior to encapsulation. hMSCs were then trypsinized, pelleted and resuspended into growth media to a desired concentration such that the final density of encapsulated hMSCs was 1

million cells per milliliter. For non-relaxing gels, stock solutions of octa-arm PEG-azide, azido-GRGDS, and the hMSC suspension were gently mixed, and then the octa-arm PEG-DBCO solution was quickly added. The mixture was pipetted several times and quickly transferred to a syringe barrel. After 10 min, the construct was equilibrated in pre-warmed media. For relaxing gels, stock solutions of octa-arm PEG-DBCO, azido-GRGDS, and the cell suspension were gently mixed. The mixture was transferred to a syringe barrel. Then octa-arm PEG-azide/FPBA and octa-arm PEG-azide/ND were sequentially added. The mixture was quickly triturated several times after adding each component. Constructs were allowed to gel at 37°C for 15 min, and were then placed into pre-warmed media. The final concentration for all components in non-relaxing and relaxing gels are shown in Table S2. Media was changed the first day after encapsulation and every other day afterwards.

Live/Dead Assay

Hydrogels were incubated in phenol red free DMEM media (supplemented with 10% FBS) with 0.5 μM calcein AM and 4.0 μM ethidium homodimer for 30 min per manufacturer's protocol. Hydrogels were imaged using an Operetta high-content imaging system. Viability was determined using this membrane integrity assay, and by counting live cells (green) and dead cells (red) in images stacked from maximum intensity projections (step size 5 μm, total thickness ~ 200 μm).

Immunostaining

Samples were fixed in 10% buffered formalin for 1 h at room temperature, washed with DPBS twice, and then permeabilized with 0.1% (v/v) Triton X-100 in PBS for 2 h at room temperature. Subsequently, constructs were incubated with 5% (w/v) BSA in PBS to block nonspecific binding, after which primary antibodies anti-YAP (mouse monoclonal, Santa Cruz Biotechnology, sc-101199, dilution of 1:250; note that this antibody was reported to stain both YAP and TAZ) in 5% (w/v) BSA were added to the constructs and incubated at 4°C overnight. Hydrogels were then rinsed with 0.5% (v/v) Tween-20 in PBS five times. Secondary antibody goat anti-mouse AlexaFluor® 647 (Invitrogen, dilution of 1:300), rhodamine phalloidin (Invitrogen, dilution of 1:300), and 4',6-diamidino-2-phenylindole (DAPI, sigma, 3.3 μg mL^{-1}) were added to the constructs and incubated at 4°C overnight. Samples were finally rinsed with 0.5% (v/v) Tween-20 in PBS five times and stored in DPBS before imaging.

Image acquisition and analysis

Immunofluorescence images were taken using a laser scanning confocal microscope (Zeiss, LSM710) with a Plan-Apochromat 20× (NA = 1.0) water objective. Images were quantified using Imaris software (Bitplane). Three dimensional views were first reconstructed from z-stack images (step size 1.5 μm). The cell and the nucleus were determined from the rhodamine and the DAPI channels, respectively (Figure S15). The volume and surface area of cell and nucleus are obtain from the Imaris software after 3D reconstruction.

The cell sphericity ψ is defined as the ratio of the surface area of a sphere with the same volume as the given cell to the surface area of the cell. The mathematical form is

$$\Psi = \frac{A_{\mathrm{sphere}}}{A_{\mathrm{cell}}} = \frac{\pi^{1/3}(6V_{\mathrm{cell}})^{2/3}}{A_{\mathrm{cell}}} \quad \text{(S18)}$$

The value of sphericity ψ ranges from 0 (a 2D object) to 1 (a perfect sphere). The nuclear sphericity is defined similarly.

The YAP/TAZ nuclear to cytosolic ratio was calculated based on the following formula,

$$\mathrm{YAP/TAZ\,nuc/cyt\,ratio} = \frac{I_{\mathrm{nuc}}/V_{\mathrm{nuc}}}{\left(I_{\mathrm{cell}} - I_{\mathrm{nuc}}\right)/\left(V_{\mathrm{cell}} - V_{\mathrm{nuc}}\right)} \qquad \text{(S19)}$$

where I_{nuc} and I_{cell} are the total intensities of the YAP/TAZ signal inside the nucleus and the cell, respectively, and V_{nuc} and V_{cell} are the volumes of the nucleus and the cell, respectively.

Statistical analysis

Experiments were performed with three independent hydrogels, and more than 30 cells were analyzed per gel. One-way analysis of variance (ANOVA) tests were performed on all data sets, followed by Tukey post-hoc tests. Tukey box-whisker plots of single cell data contained boxes corresponding to the 25th percentile and the 75th percentile. Significance levels were indicated by *, **, *** and **** for $p < 0.05$, 0.01, 0.001, and 0.0001, respectively.

Additional Supporting Figures

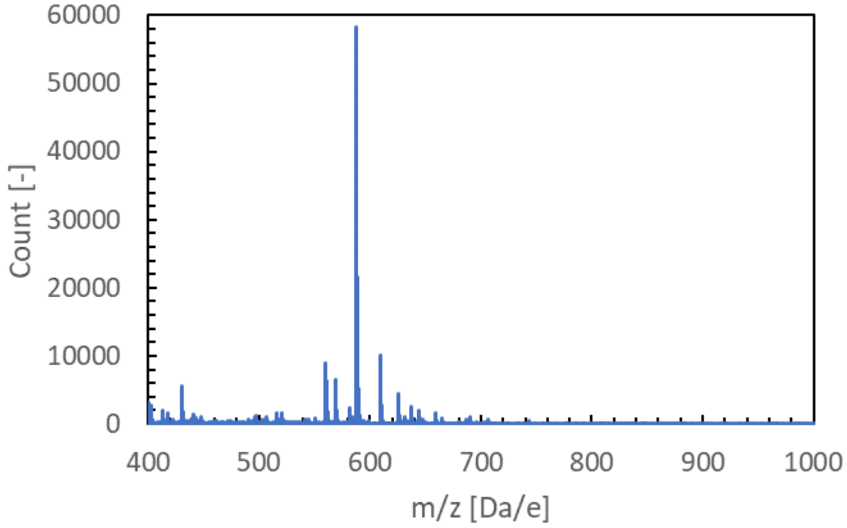

Figure S14. MALDI-TOF MS of azido GRGDS.

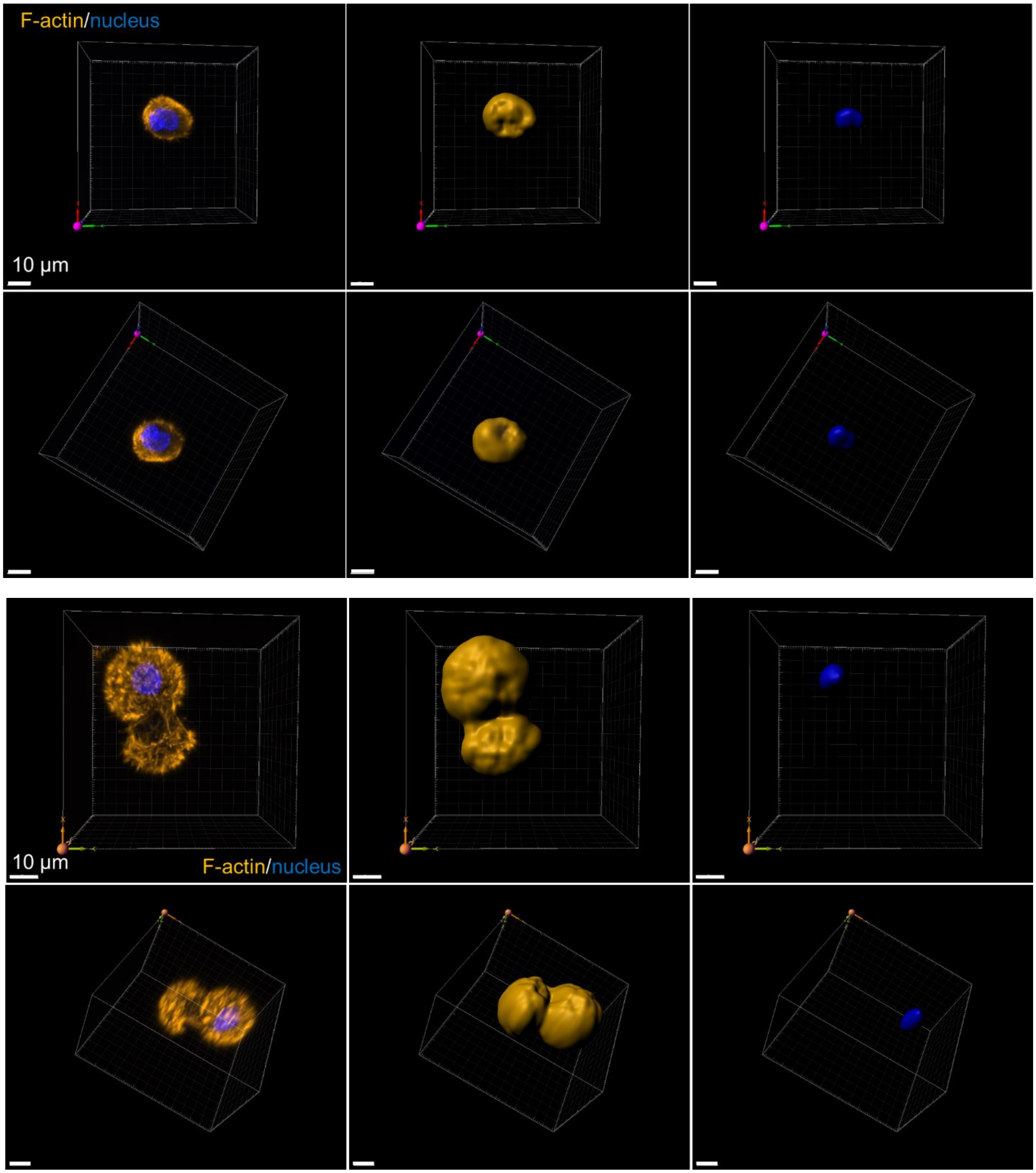

Figure S15. Representative images from 3D reconstruction.

NMR spectra

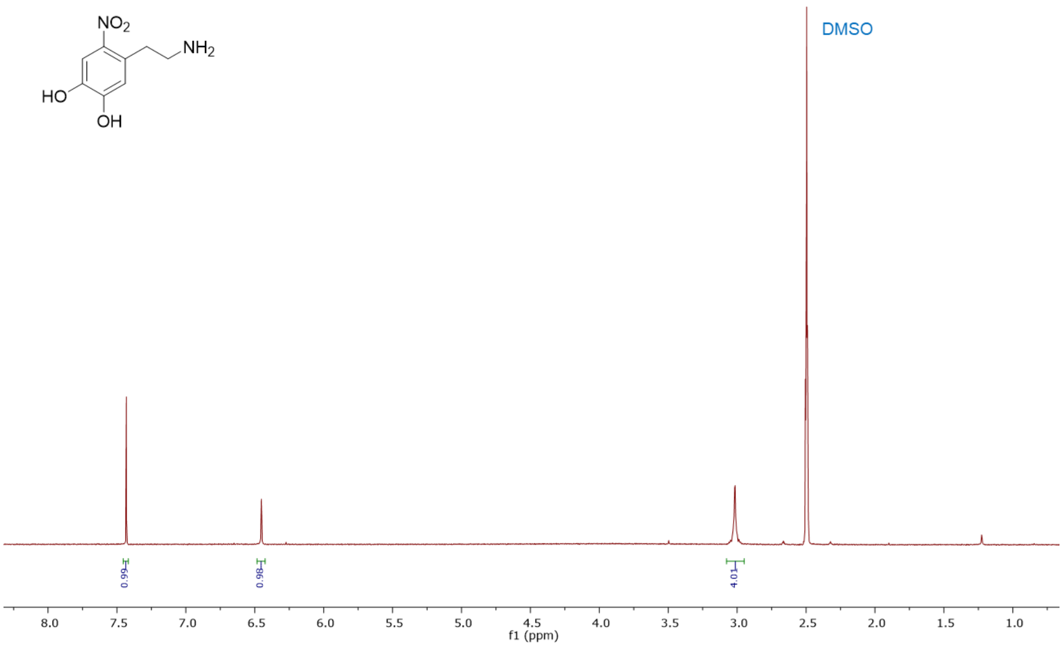

Figure S16. ^{1}H NMR spectrum of nitrodapamine (400 MHz, DMSO-d_6)

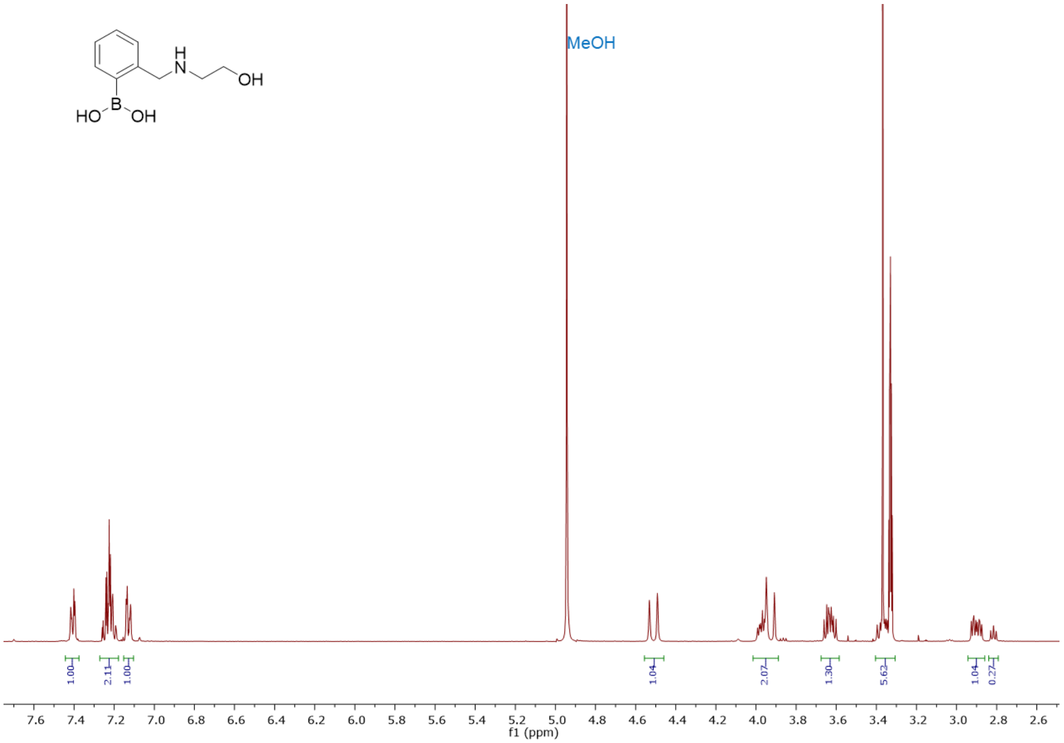

Figure S17. ^{1}H NMR spectrum of hydroxyethylaminomethylphenylboronic acid (400 MHz, MeOD)

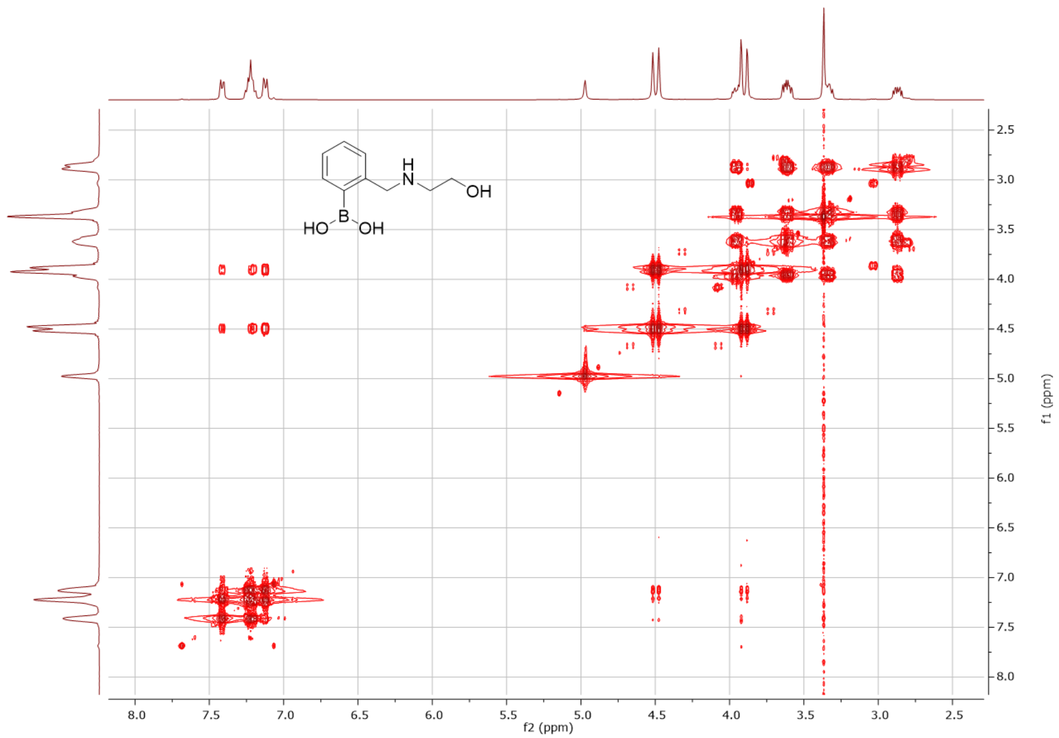

Figure S18. gCOSY spectrum of hydroxyethylaminomethylphenylboronic acid (400 MHz, MeOD)

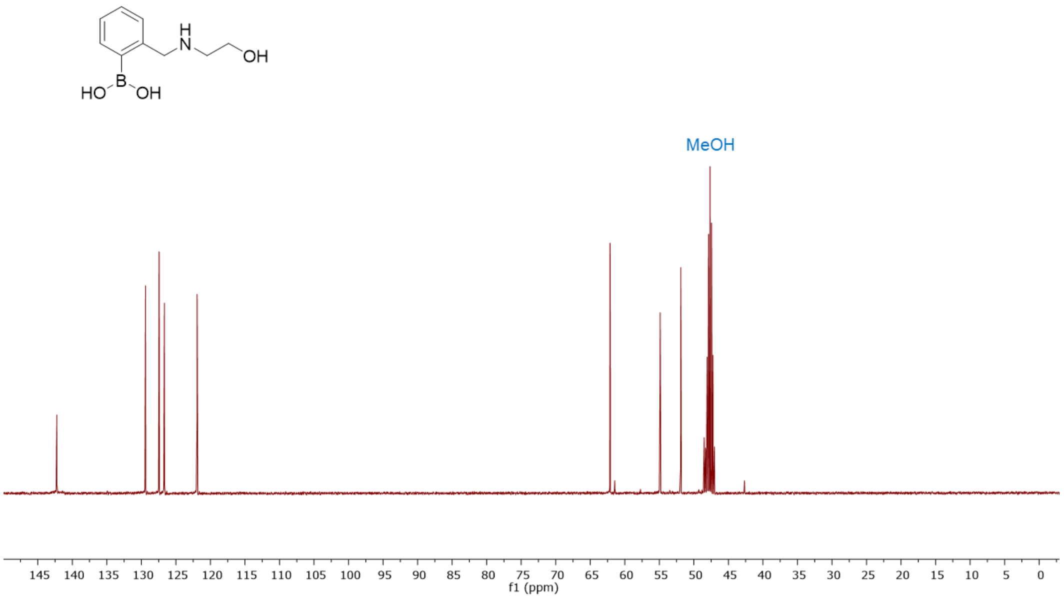

Figure S19. ^{13}C NMR spectrum of hydroxyethylaminomethylphenylboronic acid (100 MHz, MeOD)

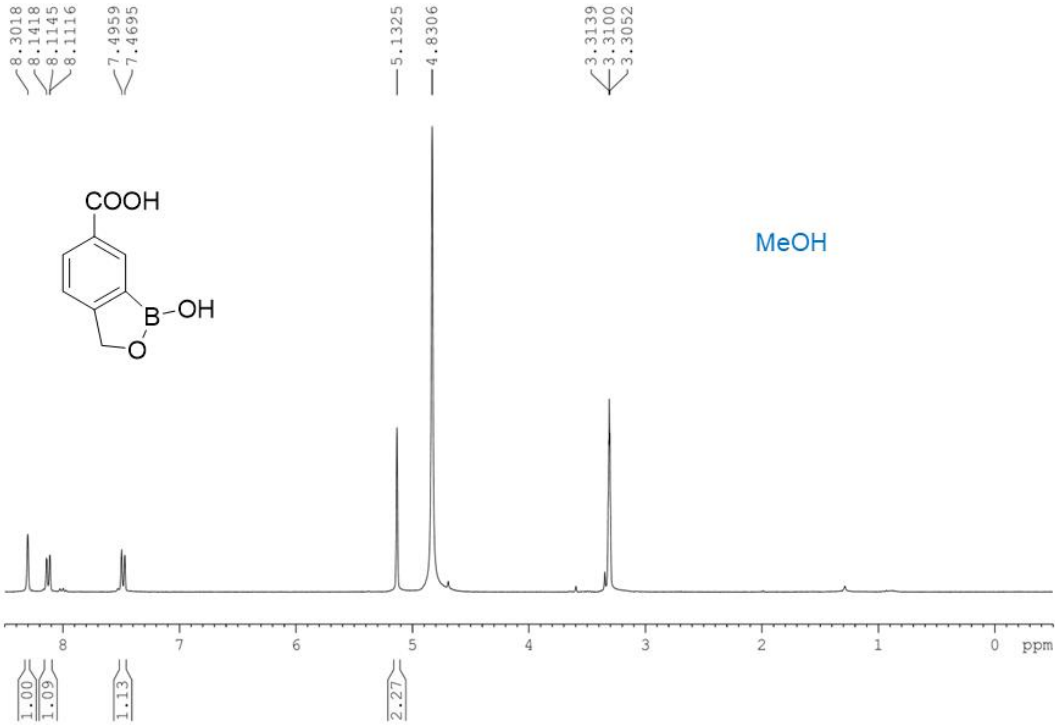

Figure S20. ^{1}H NMR spectrum of boroxole carboxylic acid (300 MHz, MeOD)

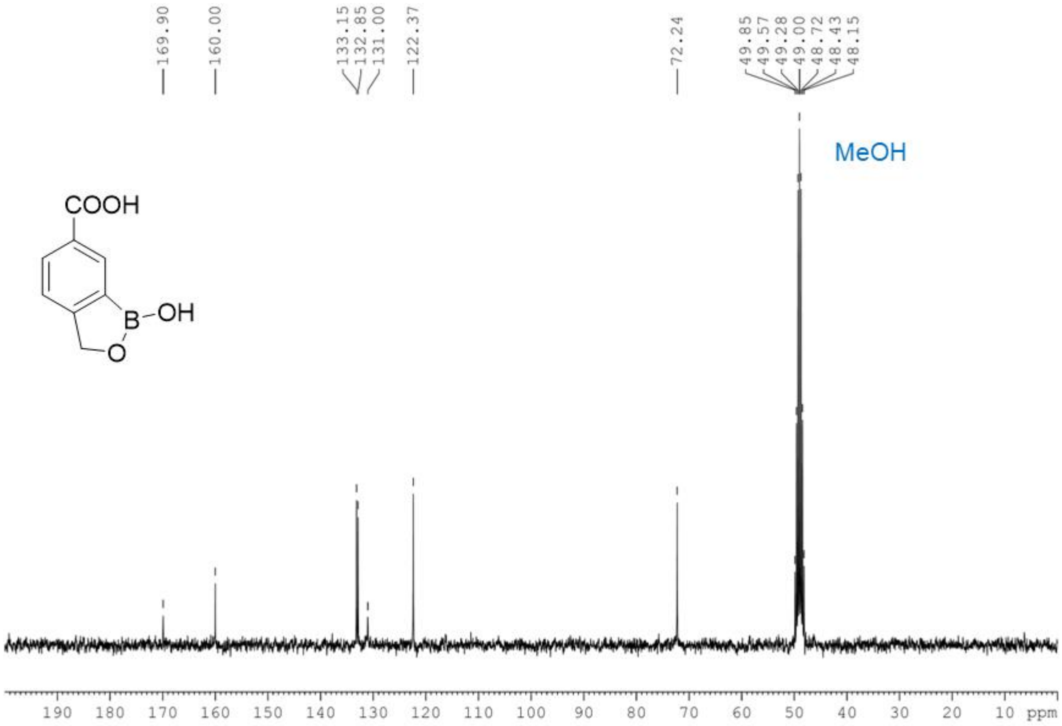

Figure S21. ^{13}C NMR spectrum of boroxole carboxylic acid (75 MHz, MeOD)

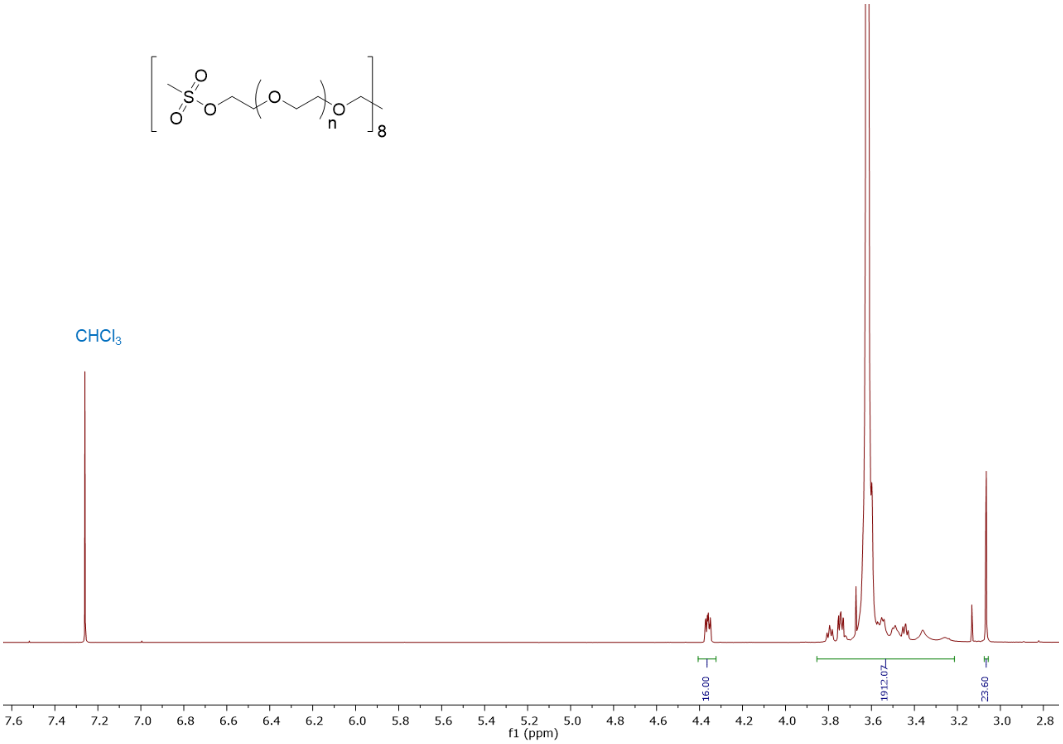

Figure S22. ^{1}H NMR spectrum of octa-arm PEG-mesylate (400 MHz, $CDCl_3$).

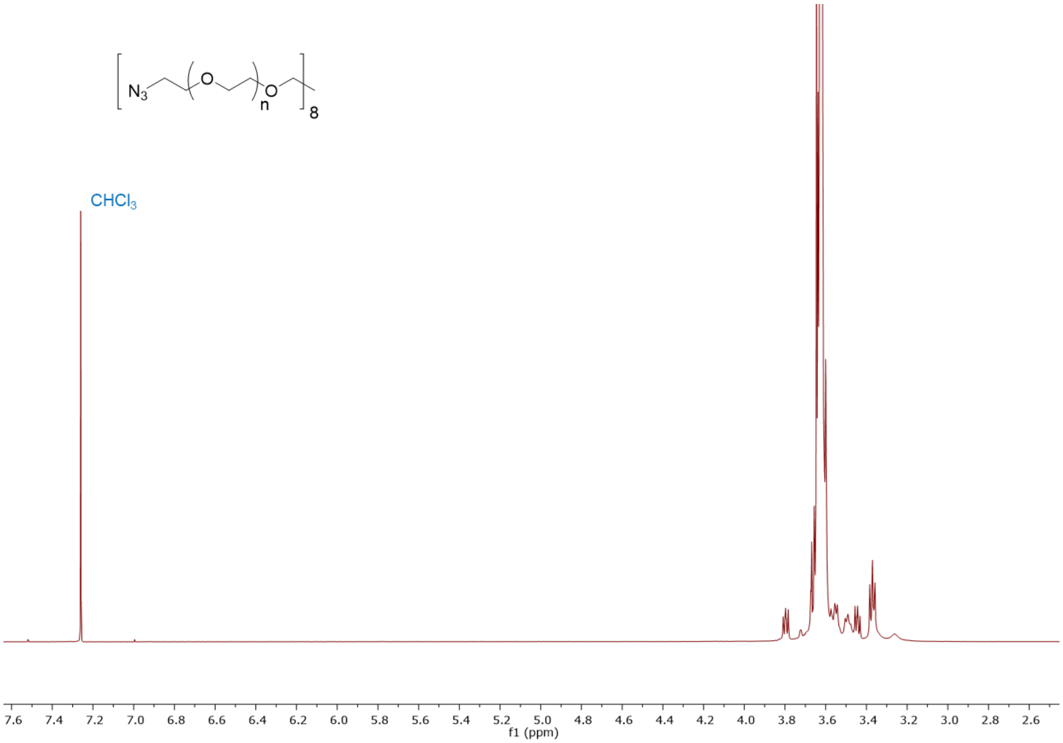

Figure S23. ^{1}H NMR spectrum of octa-arm PEG-azide (400 MHz, $CDCl_3$).

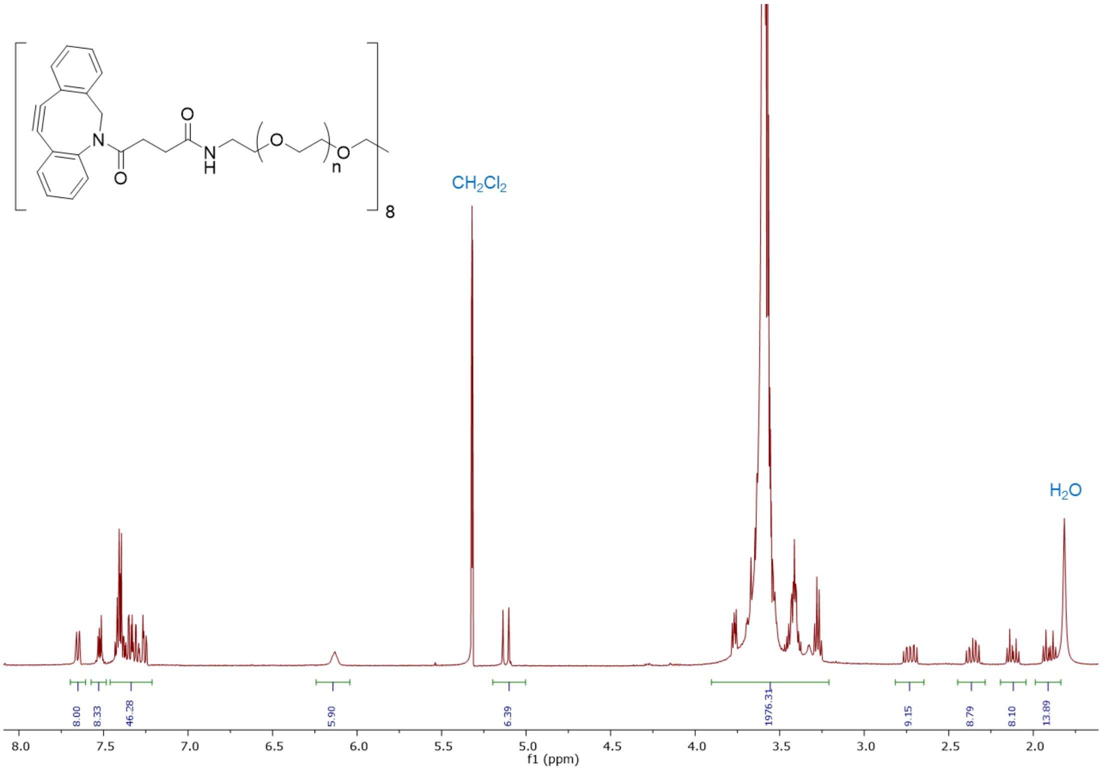

Figure S24. ^{1}H NMR spectrum of octa-arm PEG-DBCO (400 MHz, CD_2Cl_2).

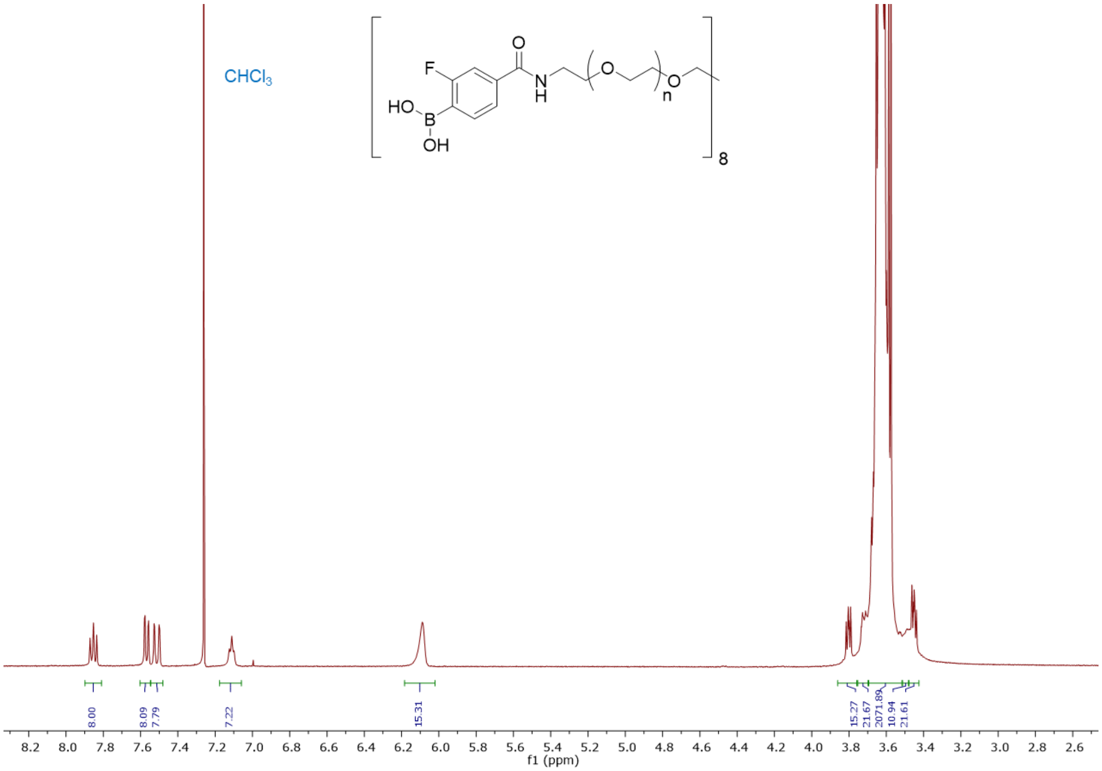

Figure S25. ^{1}H NMR spectrum of octa-arm PEG-2-fluoropehnylboronic acid (400 MHz, $CDCl_3$).

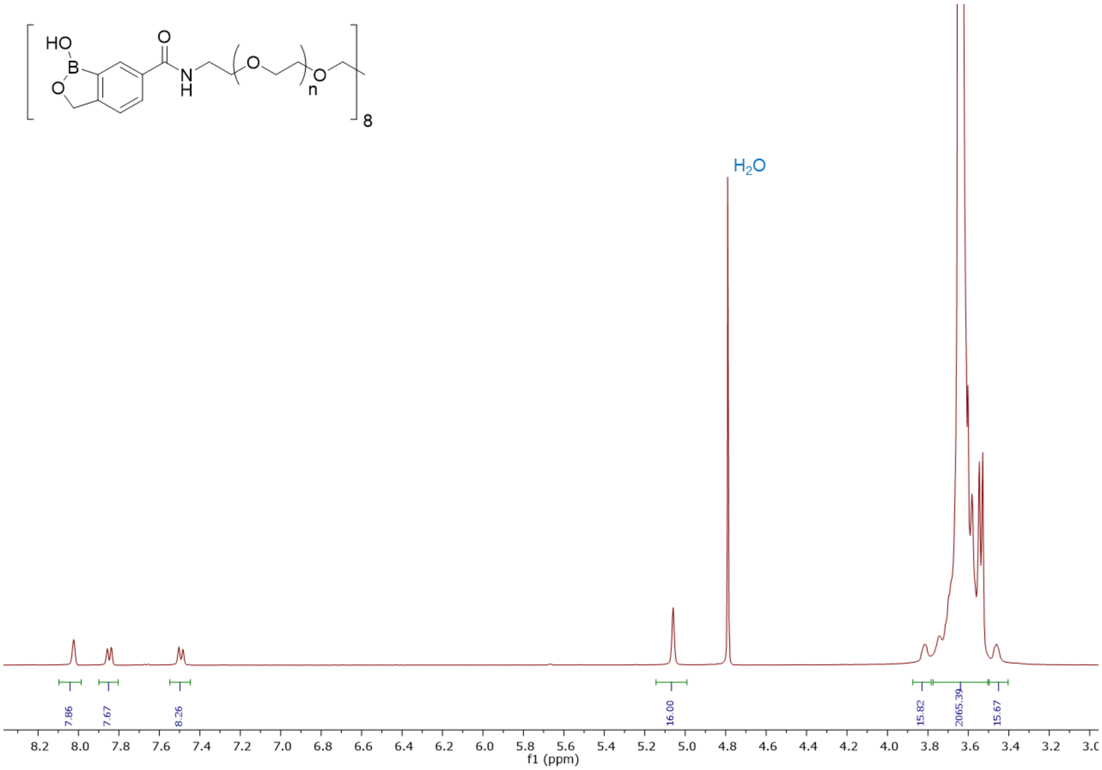

Figure S26. ^{1}H NMR spectrum of octa-arm PEG-boroxole (400 MHz, D_2O).

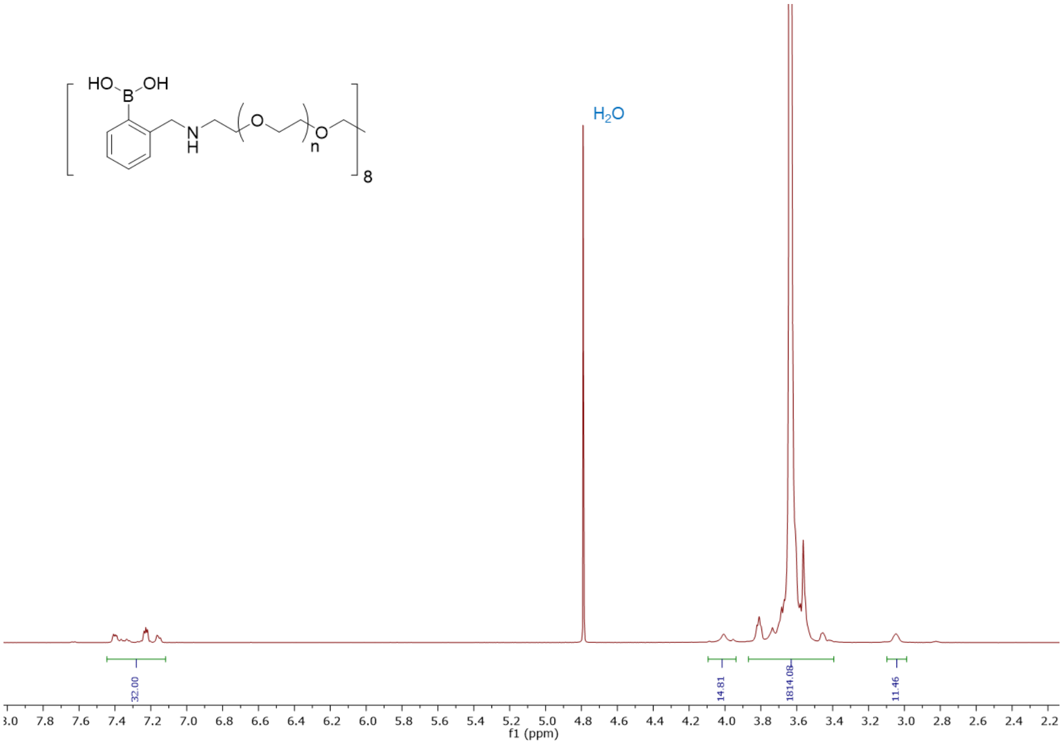

Figure S27. ^{1}H NMR spectrum of octa-arm PEG-o-aminomethylphenylboronic acid (400 MHz, D_2O).

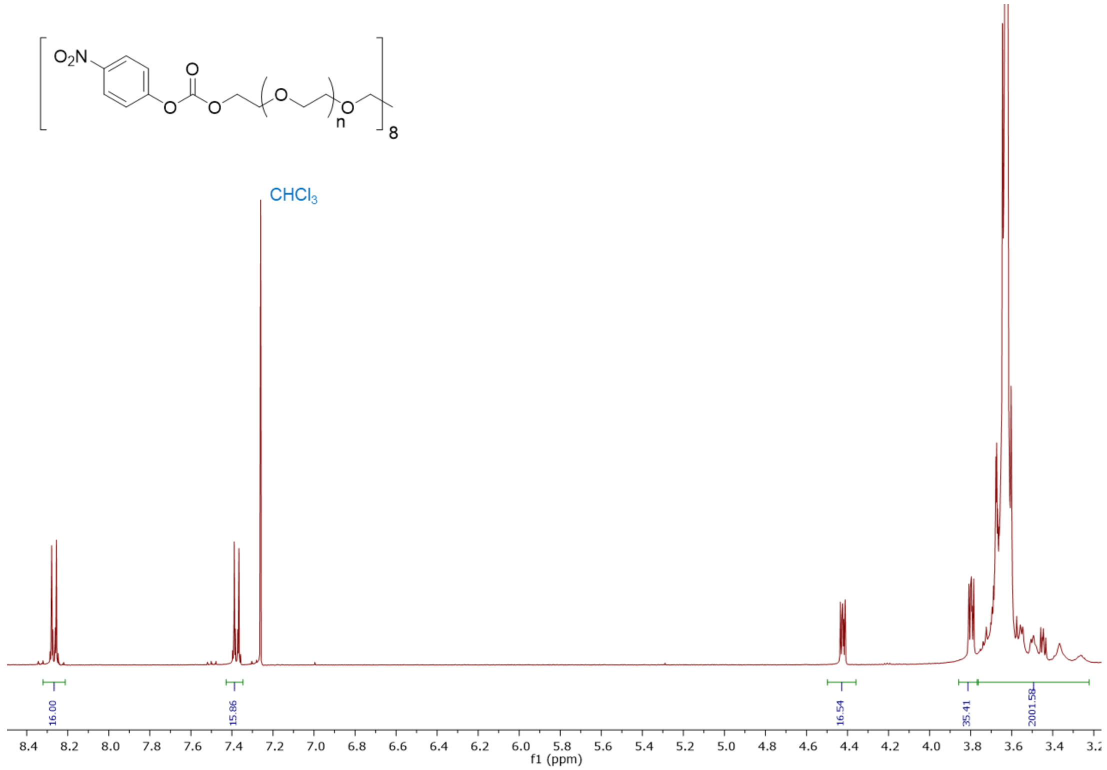

Figure S28. ^{1}H NMR spectrum of octa-arm PEG-*p*-nitrophenylcarbonate (400 MHz, $CDCl_3$).

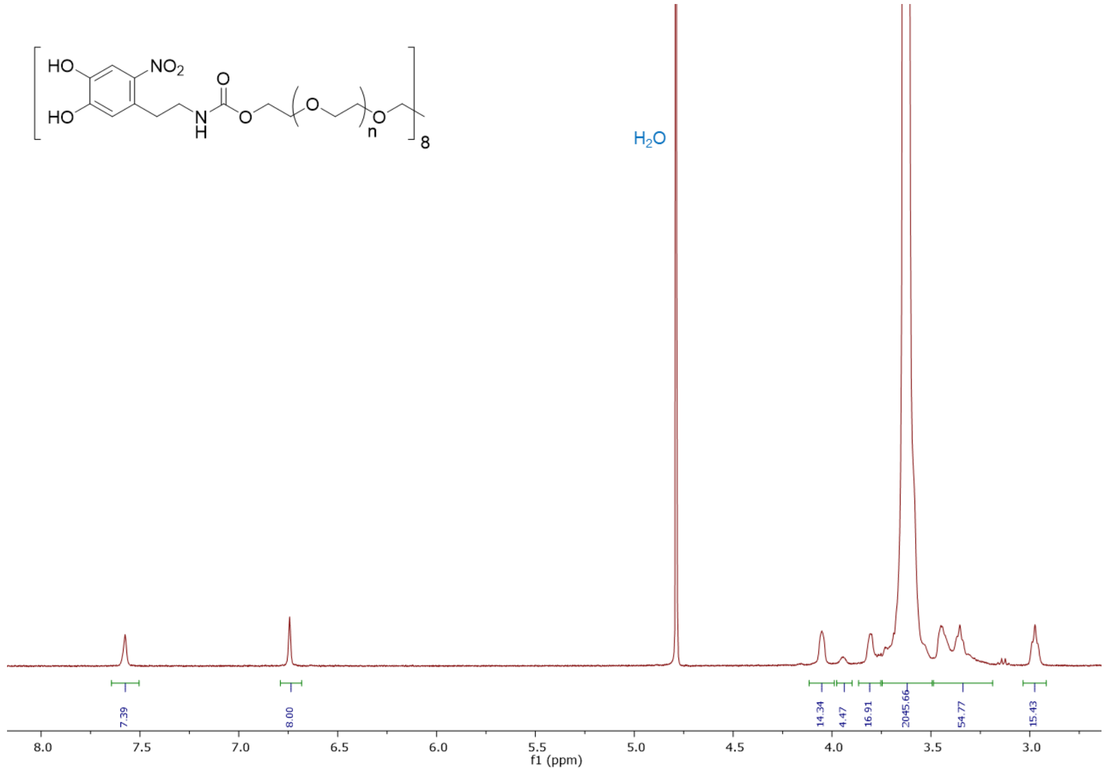

Figure S29. ^{1}H NMR spectrum of octa-arm PEG-nitrodopamine (400 MHz, D_2O).

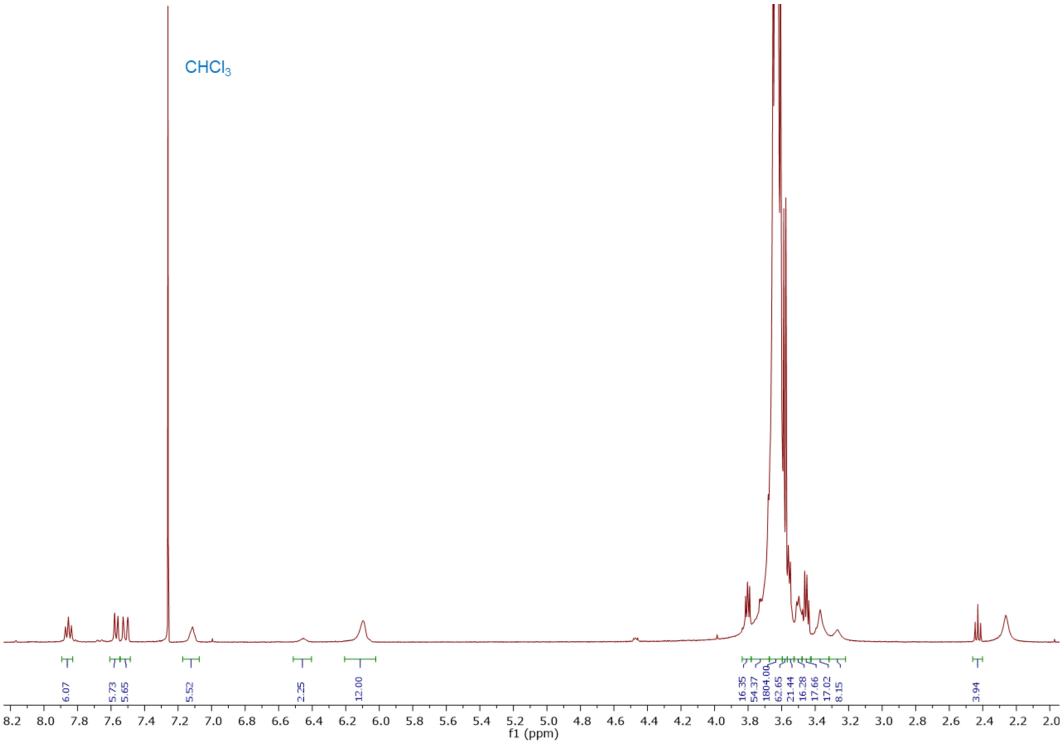

Figure S30. ^{1}H NMR spectrum of octa-arm PEG-azide/fluorophenylboronic acid (400 MHz, $CDCl_3$).

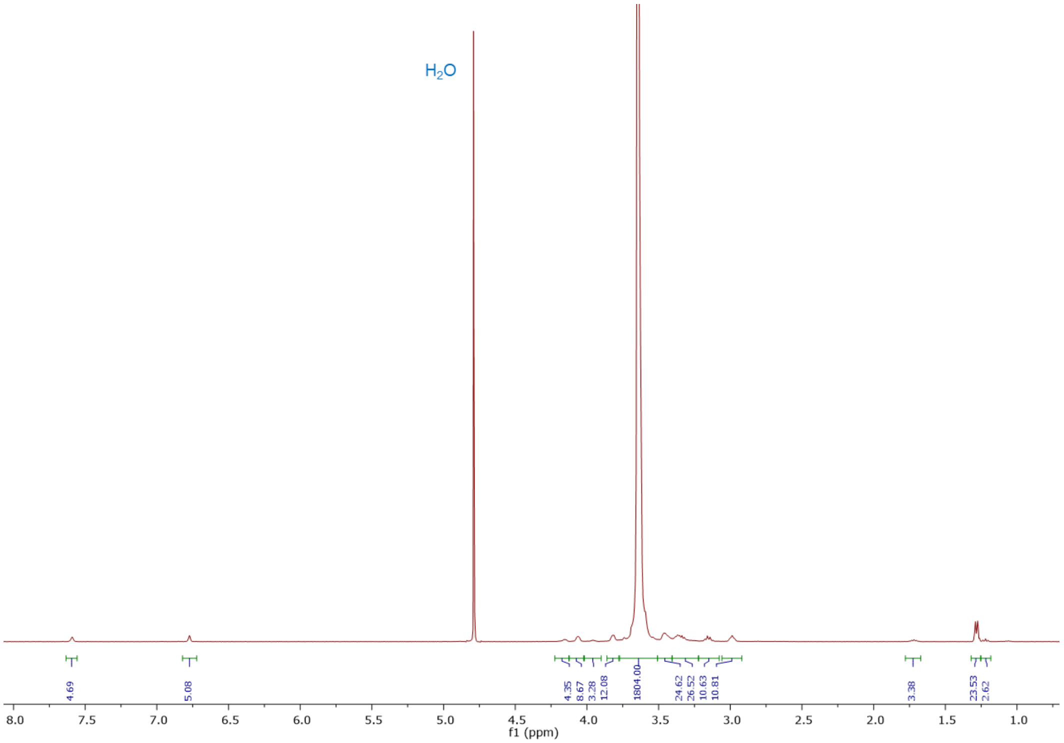

Figure S31. ^{1}H NMR spectrum of octa-arm PEG-azide/nitrodopamine (400 MHz, D_2O).

References

[1] Z. Shafiq, J. Cui, L. Pastor-Pérez, V. San Miguel, R. A. Gropeanu, C. Serrano, A. Del Campo, *Angew. Chemie - Int. Ed.* **2012**, *51*, 4332.

[2] O. R. Cromwell, J. Chung, Z. Guan, *J. Am. Chem. Soc.* **2015**, *137*, 6492.

[3] I. N. Houpis, J.-P. V. Hoeck, U. Tilstam, *Synlett* **2007**, 2179.

[4] K. T. Kim, J. J. L. M. Cornelissen, R. J. M. Nolte, J. C. M. van Hest, *J. Am. Chem. Soc.* **2009**, *131*, 13908.

[5] H. Kim, Y. J. Kang, S. Kang, K. T. Kim, *J. Am. Chem. Soc.* **2012**, *134*, 4030.

[6] C. Z. Ding, Y.-K. Zhang, X. Li, Y. Liu, S. Zhang, Y. Zhou, J. J. Plattner, S. J. Baker, L. Liu, M. Duan, R. L. Jarvest, J. Ji, W. M. Kazmierski, M. D. Tallant, L. L. Wright, G. K. Smith, R. M. Crosby, A. A. Wang, Z.-J. Ni, W. Zou, J. Wright, *Bioorg. Med. Chem. Lett.* **2010**, *20*, 7317.

[7] T. Rossow, S. Seiffert, *Polym. Chem.* **2014**, *5*, 3018.

[8] V. Yesilyurt, A. M. Ayoob, E. A. Appel, J. T. Borenstein, R. Langer, D. G. Anderson, *Adv. Mater.* **2017**, *29*, DOI 10.1002/adma.201605947.

[9] S. Lascano, K.-D. Zhang, R. Wehlauch, K. Gademann, N. Sakai, S. Matile, *Chem. Sci.* **2016**, *7*, 4720.

[10] J. Yan, G. Springsteen, S. Deeter, B. Wang, *Tetrahedron* **2004**, *60*, 11205.

[11] S. Tang, M. Wang, B. D. Olsen, *J. Am. Chem. Soc.* **2015**, *137*, 3946.

[12] J. D. Ferry, *Viscoelastic Properties of Polymers*, **1980**.

[13] J. Brandrup, E. Immergut, E. A. Grulke, *John Wiley Sons, Inc* **1990**, *12*, 265.

[14] P. D. Mariner, E. Johannesen, K. S. Anseth, *J. Tissue Eng. Regen. Med.* **2012**, *6*, 314.
